# Seasonal Variation in Shell Calcification of Planktonic Foraminifera in the NE Atlantic Reveals Species-Specific Response to Temperature, Productivity, and Optimum Growth Conditions

**DOI:** 10.1371/journal.pone.0148363

**Published:** 2016-02-09

**Authors:** Manuel F. G. Weinkauf, José G. Kunze, Joanna J. Waniek, Michal Kučera

**Affiliations:** 1 Department of Geosciences, Eberhard–Karls University, Tübingen, Germany; 2 MARUM, University Bremen, Bremen, Germany; 3 Marine Chemistry Section, Leibniz Institute for Baltic Sea Research Warnemünde, Rostock, Germany; Auckland University of Technology, NEW ZEALAND

## Abstract

Using shells collected from a sediment trap series in the Madeira Basin, we investigate the effects of seasonal variation of temperature, productivity, and optimum growth conditions on calcification in three species of planktonic Foraminifera. The series covers an entire seasonal cycle and reflects conditions at the edge of the distribution of the studied species, manifesting more suitable growth conditions during different parts of the year. The seasonal variation in seawater carbonate saturation at the studied site is negligible compared to other oceanic regions, allowing us to assess the effect of parameters other than carbonate saturation. Shell calcification is quantified using weight and size of individual shells. The size–weight scaling within each species is robust against changes in environmental parameters, but differs among species. An analysis of the variation in calcification intensity (size-normalized weight) reveals species-specific response patterns. In *Globigerinoides ruber* (white) and *Globigerinoides elongatus*, calcification intensity is correlated with temperature (positive) and productivity (negative), whilst in *Globigerina bulloides* no environmental forcing is observed. The size–weight scaling, calcification intensity, and response of calcification intensity to environmental change differed between *G. ruber* (white) and *G. elongatus*, implying that patterns extracted from pooled analyses of these species may reflect their changing proportions in the samples. Using shell flux as a measure of optimum growth conditions, we observe significant positive correlation with calcification intensity in *G. elongatus*, but negative correlation in *G. bulloides*. The lack of a consistent response of calcification intensity to optimum growth conditions is mirrored by the results of shell size analyses. We conclude that calcification intensity in planktonic Foraminifera is affected by factors other than carbonate saturation. These factors include temperature, productivity, and optimum growth conditions, but the strength and sign of the relationships differ among species, potentially complicating interpretations of calcification data from the fossil record.

## Introduction

Planktonic Foraminifera are important marine calcifiers, contributing 30–80% to the global pelagic carbonate flux [1]. Considering the importance of planktonic Foraminifera for the global carbon cycle, the processes controlling how much calcite is secreted during the life of an individual remain poorly constrained. Calcification in planktonic Foraminifera is an energy-consuming process [2, 3], making it likely that it participates in trade-offs of energy allocation within the cell. Thus, in theory, several environmental parameters, such as seawater carbonate saturation or ambient temperature, could influence calcification. The distance from the ecological optimum of a species could also be important, as it can affect how much energy is available for calcification under sub-optimal living conditions.

Most often, calcification intensity in planktonic Foraminifera (i.e. amount of calcite normalized for shell size) has been correlated with the physical and chemical properties of their environment. However, there is broad disagreement about the dominant controlling parameters and even about the nature of their relationship with calcification. Based on field observations [4–9] and laboratory culturing studies [10, 11], carbonate saturation of the ambient seawater appears to be the most promising parameter to explain variations in the calcification intensity of planktonic Foraminifera. However, an analysis of plankton samples from the Arabian Sea [12] revealed that the shape of the relationship between carbonate saturation and shell weight is species-specific and its sign is not always positive. Subsequently, culturing experiments [13] have shown that the effect of seawater carbonate chemistry on shell calcification in Foraminifera is also moderated by temperature. Similarly, a study based on Pliocene sediments [14] also found no link between calcification in planktonic Foraminifera and atmospheric *p*CO_2_, but rather identified temperature as a potential factor explaining the observed variation in foraminiferal shell calcification. Because both parameters are tightly linked, it is challenging to disentangle their relative contributions even in well constrained studies based on recent sediment-trap material [9].

Carbonate chemistry and temperature are not the only variables invoked to explain changes in calcification of planktonic Foraminifera. In plankton material from the North Atlantic, phosphate concentration in the ambient sea water was identified as the potentially dominant factor influencing the calcification of *Globigerina bulloides* d’Orbigny, 1826 [15], but this study suffered from multicollinearity among the studied environmental parameters. Conversely, de Villiers [16] proposed that shell calcification in planktonic Foraminifera could be linked to growth under optimal environmental conditions, meaning that shell calcification is highest when the combination of all environmental factors is close to the optimum of the species. A similar relationship was suggested for the calcification of *Globigerinoides ruber* (d’Orbigny, 1839) in sediment trap samples from the Arabian Sea [17]. Also, growth under optimum conditions has been invoked as the best predictor of the overall mean shell size of specimens within species of planktonic Foraminifera [18, 19]. Assuming that optimal environmental conditions are mirrored in the absolute and relative abundances of a species (with higher abundances indicating more optimal environments), the relationship between optimum growth and calcification has been tested in fossil samples from a Mediterranean sapropel [20]. This study found no evidence for a relationship between calcification and ecological optimum, but identified changes in seawater properties as the most likely parameter affecting calcification.

A pre-requisite for any investigation of factors controlling calcification in planktonic Foraminifera is a definition of a meaningful measure of the amount of calcite precipitated by one individual. This quantity can be easily determined as the weight of the shell [21], but it reflects two parameters: calcification intensity and shell size. To use shell weight as a proxy for calcification intensity, one could thus either normalize weight by size or determine the weight of shells of equal sizes. Traditionally, calcification intensity in planktonic Foraminifera has been quantified by using a parameter known as size-normalized weight (SNW), which is a compromise between the two possible strategies. Its most simple form is the sieve-based weight (SBW) [4, 5, 22] where multiple individuals in a narrow size fraction are weighed together and then the mean of their weight is determined. A more advanced version is the measurement-based weight (MBW) [6, 15], where the SBW is normalized for the actual measured mean individual shell size of the specimens in the weighed size fraction. In theory, when the actual size of the measured individuals is determined, the measure of calcification intensity does not have to be limited to a narrow size fraction and the calcification of an individual shell can be directly normalized to its size, typically approximated by the cross-sectional area of the shell (area density, AD) [9] and then averaged per sample.

**Fig 1:**
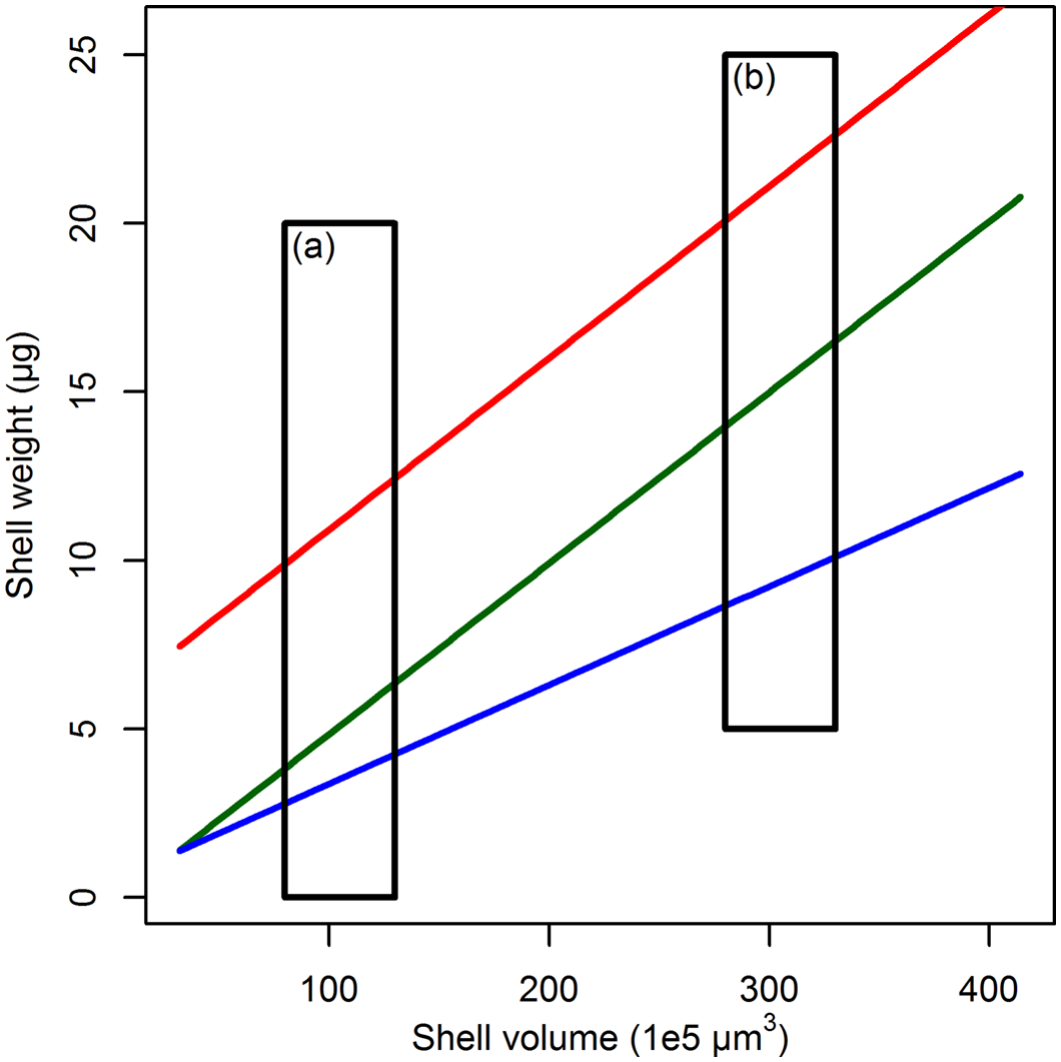
Conceptual figure illustrating the difficulties of interpreting the size-normalized weight when the size–weight scaling is not constant. Lines depict the size–weight scaling from three hypothetical communities (color-coded), boxes (a) and (b) are two possible samples from restricted size fractions of those communities. Within the green and the red assemblage, the scaling remains constant at 0.05 µg/1 × 10^−5^ µm^3^. Here, the chosen size fraction for measuring the calcification intensity does not influence the results, since the offset between both lines is constant for all shell sizes. The blue assemblage, however, shows a lower scaling slope of only 0.03 µg/1 × 10^−5^ µm^3^. Here the observed difference in calcification intensity with both the green and red assemblage would be larger in size fraction (b) than they are in (a).

All of these approaches make the critical assumption that calcification intensity is independent of shell size. In plankton samples they also assume that the measured specimens all represent an equivalent ontogenetic stage, because calcification increases with ontogeny [23] and renders data from plankton samples potentially difficult to interpret. In sediment trap samples and in the sediment, the majority of the deposited shells represent adult individuals that have undergone the same ontogenetic pathway [24]. However, sedimentary individuals attributable to the same species vary in size considerably and it has never been established how calcification intensity scales across the analyzed range of shell sizes. Until now, all studies have assumed that calcification intensity is invariant to size and considered “mean” calcification intensity within one size range to be representative for all individuals in the analyzed population, although this assumption has been debated before [25]. If calcification intensity varies with size (e.g. because large shells have a higher calcification efficiency), averaged biometric data that are not restricted to small size fractions could bias the interpretation of calcification intensity (Fig 1). This assumption can be easily tested by determining the relationship between weight and size for individual shells across a range of sizes. If there is no change in calcification intensity with size, the relationship will be linear (as long as size is scaled to volume), the residuals will be small, and the slope of the linear regression will be the same across all samples studied.

**Fig 2:**
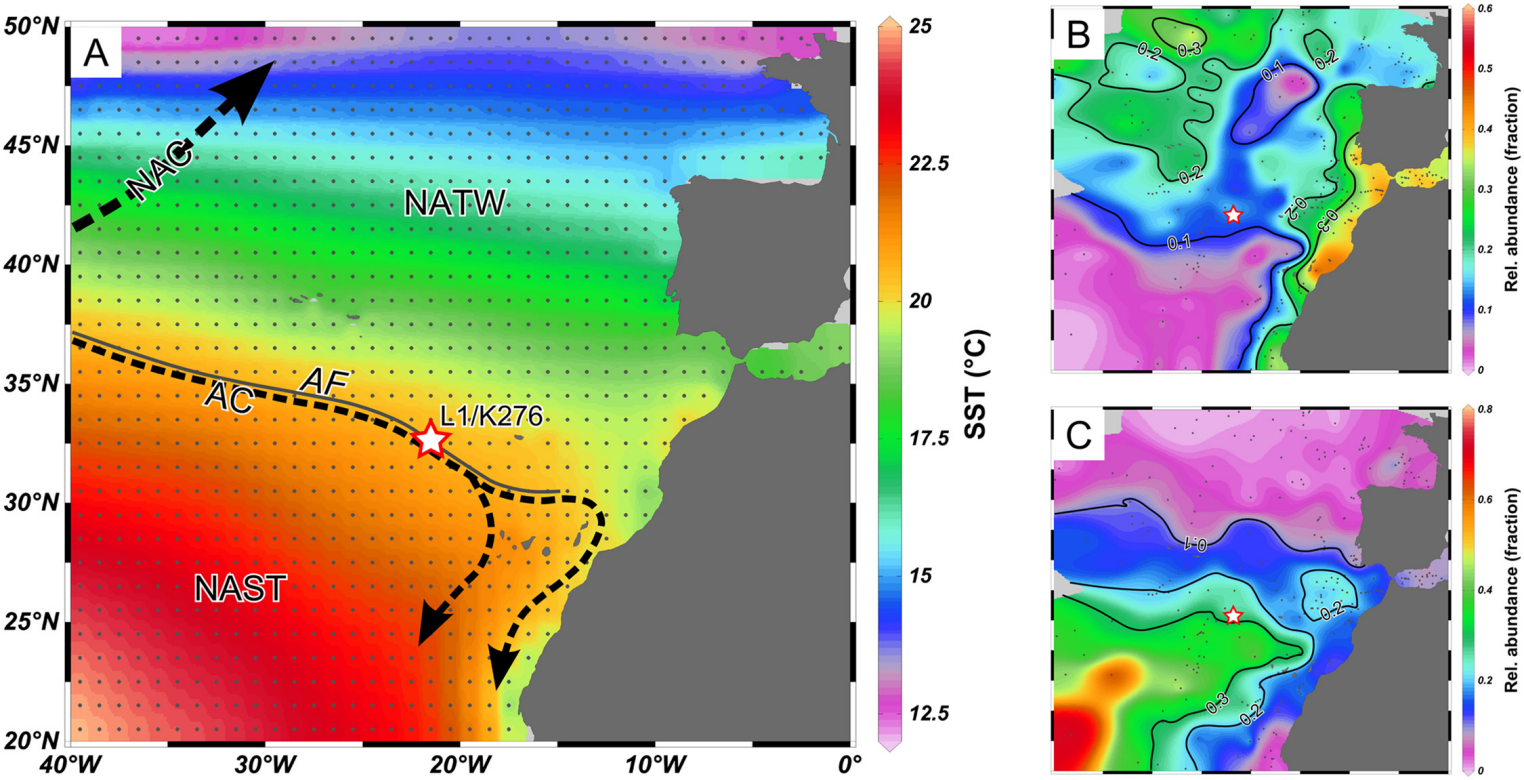
Regional setting and abundance pattern (MARGO database) [35, 36] of investigated species around sediment trap L1/K276 (red–white star), plotted with Ocean Data View v. 4.6.2 [37]. Original data points used for interpolation are indicated with gray dots, light gray indicates areas with no data. (A) Annual mean sea surface temperature (SST) [28] and main ocean currents of the region. The trap is situated in the direct vicinity of the Azores Front (AF) and Azores Current (AC). The Azores Front separates the North Atlantic Transitional Water (NATW) with mean SST below 20°C from the North Atlantic Subtropical Gyre (NAST) with a mean SST above 20°C. NAC = North Atlantic Current. (B) *Globigerina bulloides* shows local abundances between 10 and 20% in the area, which are increasing northward. (C) Mean abundances of the *G. ruber/elongatus* plexus (which are not separated in MARGO) are approximately 30%, and are increasing to the south. The sediment trap is thus positioned at the edge of the regional distribution area of all three species.

This study therefore aims at specifically testing the stability of the relationship between calcification intensity and size across species and under different environmental conditions, and at contributing to the understanding of factors other than carbonate saturation affecting calcification intensity in planktonic Foraminifera. Sediment samples integrate shell flux over several years, so that shell size and weight relationships within the sample can not be directly attributed to forcing, and plankton material will be dominated by the ontogenetic process, bearing little relevance for studies of the fossil record. Therefore, we consider sediment trap samples as optimum choice for such an analysis, because they provide both high temporal resolution with adequate sample sizes and a limitation of the sample to sinking adult specimens equivalent to those found in the sediment. Specifically, we use material collected with a sediment trap in the North Atlantic Madeira Basin close to the Azores Front (Fig 2A). The Azores Front is situated in the northeastern Atlantic, resulting from the Azores Current that flows towards east-south-east as a branch of the North Atlantic Current [26]. The Azores Front is separating the cooler regions to the north from the warm North Atlantic Subtropical Gyre [27] to the south [28]. Due to the seasonal variability in the position of the Azores Front and Azores Current [29], the catchment area of the studied sediment trap shows a large seasonal cycle in surface water conditions. The local sea surface temperature ranges between ca. 17–18°C early in the year and ca. 24–25°C during late summer and autumn, with eutrophic conditions during January–March and an oligotrophic summer and autumn [30]. While no actual data for the seawater carbonate chemistry in our study exist, we could use average data for temperature [28], salinity [31], phosphate and silicate content [32], and total CO_2_ and alkalinity [33] to calculate the average seasonal variation of [CO_3_^2−^] during the year (S1 Table). The average seasonal carbonate saturation in the catchment area of the sediment trap [30] ranges from 213.9 µmol kg^−1^ in spring to less than 215.2 µmol kg^−1^ in winter and summer, and only reaches peak values of 220.3 µmol kg^−1^ in autumn (calculated with CO2Sys, MS Excel v. 2.1) [34]. The regional carbonate system is nearly exclusively influenced by temperature, with salinity only contributes to slightly more than 10% to the total carbonate saturation change.

The average three-weeks sampling resolution assures that each sample represents the deposition of one or very few generations of Foraminifera [38], which were exposed to near-constant environmental conditions. Because the sampling covers one entire seasonal cycle, we can investigate how the size–weight relationship in multiple species behaves in regard to changing environmental conditions. Varying on average by no more than 7 µmol kg^−1^, the carbonate saturation at the studied locality is nearly constant, allowing us to study the effects of temperature and ecological optimum independent of carbonate chemistry. Using this natural experiment, we can use measurements of individual shell size and weight to determine the stability of the size–weight relationship and assess the effect of several environmental factors on the calcification intensity of planktonic Foraminifera whilst accounting for that effect.

## Materials and Methods

### Sample Collection and Preparation

This study is based on material collected by the JGOFS trap 53 from station L1/K276, located around 33° N and 22° W in the Madeira Basin, in direct vicinity to the Azores Front, with a local ocean depth of approximately 5500 m at the mooring (Fig 2). The trap has been deployed at about 2000 m depth, sampling between February 2002 and April 2003 with variable sampling duration (ranging between 6 and 61 days), adapted to the expected seasonal particle flux. Special sampling permissions were not required because the sediment trap was located in international waters. We did not sample any endangered or protected species for this study. A total of 18 sample cups were used for our study. Information on trap design, sample treatment, and physical oceanography during deployment are reported in Waniek et. al. [30] and Fründt and Waniek [29]; sample processing for analysis of planktonic Foraminifera assemblage composition is described in Storz et al. [39]. Only the fraction >150 µm (separated by dry-sieving) was used for this study, because using this size fraction ensures that the majority of specimens represent adult individuals [40].

### Choice of Species

**Fig 3:**
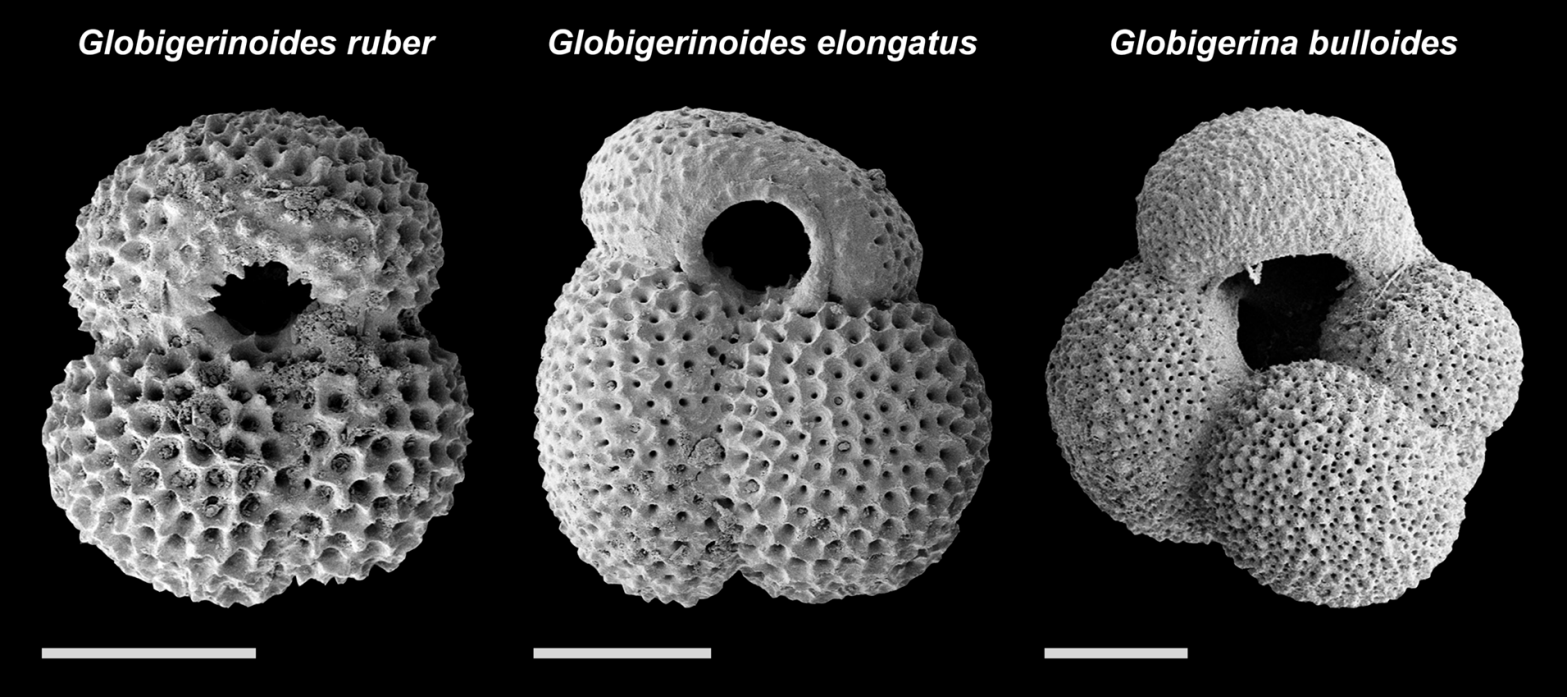
Scanning electron microscope images of specimens of the three species of planktonic Foraminifera used in this study, representative for the respective morphology: *G. ruber* (Sample 11), *G. elongatus* (Sample 9), and *G. bulloides* (Sample 3). Scale bars equal 100 µm in length.

The species for this study (Fig 3) were chosen to represent a broad environmental spectrum while at the same time occurring in sufficient abundances to provide suitable sample sizes. *Globigerinoides ruber* (white) (corresponding to the traditional concept of the *G. ruber* s.str. morphotype) is a symbiont bearing species that is bound to the upper water column due to the photosynthetic activity of its symbionts. It was shown to be highly abundant throughout the year, with peak abundances of up to 40% of the total community of planktonic Foraminifera between July and January [39]. The activity of the symbionts in *G. ruber* (white) may buffer environmental effects otherwise influencing the ability of the species to calcify its shell. Therefore, *Globigerina bulloides* has been selected as the second species for this study. This species does not possess symbionts, but shares a similar depth habitat with *G. ruber* (white) in the studied region (maximum abundances occur above 100 m water depth [41]). The distribution of *G. ruber* (white) and *G. bulloides* in the sediment (Fig 2B and 2C) indicates that the position of the sediment trap for both species is close to their ecological limits. *Globigerinoides ruber* (white) is a subtropical species whereas *G. bulloides* is a temperate species, as is also supported by their abundance in the trap series [39]. The morphological groups *G. ruber* s.str. (inflated chambers in the last whorl) and *G. ruber* s.lat. (compressed chambers in the last whorl) have been recognized within *G. ruber* (white) [42]. In a combined morphological and genetic investigation, it was shown that the morphotype *G. ruber* s.lat. represents a different species [43]. Following the criteria in Aurahs et al. [43], we use the name *G. ruber* (white) for specimens of the morphotype *G. ruber* s.str., and include in our analysis a third species *Globigerinoides elongatus* (d’Orbigny, 1826) that refers to specimens of the *G. ruber* s.lat. morphotype. The ecology of *G. elongatus* has not been studied in detail, but it appears that the species has a slightly broader ecological range than *G. ruber* (white) and calcifies deeper in the water column [44, 45]. It has been shown that *G. bulloides* also comprises a mixture of at least three genotypes in the study area [46], but since those cannot be distinguished on the basis of their morphology we cannot fully control for their potential influence in this study. All species chosen for this study are known to not deposit large amounts of gametogenetic calcite [47], so that in combination with using the size fraction >150 µm to ensure individuals are predominately adult, results will not be biased by varying reproduction success of the studied communities (S1 File).

### Data Acquisition

Specimens of the species *G. ruber* (white), *G. elongatus*, and *G. bulloides* were picked from the >150 µm fraction in all samples and transferred into cardboard slides for further processing. The flux of the three species was calculated by dividing their observed abundances in the >150 µm fraction by the opening area of the trap (0.5 m^2^ [48]) and by the sampling duration (in days) of the individual samples. We did not use the flux published in Storz et al. [39], because there fluxes for the fraction >125 µm are given and no distinction between *G. ruber* (white) and *G. elongatus* is made. As a result, the flux presented here is much smaller (up to 85%) than that shown in Storz et al. [39], but this is consistent with the fact that the majority of specimens in all species (on average 50% in *Globigerinoides* and 60% in *G. bulloides*) are between 125 µm and 150 µm in size [49]. When qualitatively comparing our fluxes with those from Storz et al. [39] from the same samples, a Spearman rank-order correlation shows a highly significant correlation (*p* <.001) with correlation coefficients of *ρ* > 0.8, further supporting our data.

The size of all specimens >150 µm of the three species has been determined by photographing them in umbilical view under constant magnification using a Leica Z16 stereomicroscope equipped with a 5 MPx industrial camera, and the photographs were analyzed with the Image-Pro Plus software [50]. As size parameters, the length of the longest shell-axis (Feret diameter) and the cross-sectional area of the shell were extracted from the images.

To determine the calcification intensity, all specimens of the three species within a certain size range were individually weighed. For *G. ruber* (white) and *G. elongatus*, all specimens from the 200–250 µm size range were measured, to obtain values best comparable with previous investigations [51]. For *G. bulloides* the 200–300 µm size range was used in order to obtain more individuals and to investigate the linearity of the size–weight relationship across a broader size range. Individual shells were transferred into tin weighing boats and repeatedly (4–5 times) weighed with a Mettler Toledo UMX 2 microbalance, to alleviate the effects of drift and external disturbance during the weighing process. The mean value of the repeated measurements was used as individual weight parameter per shell, which allowed to calculate the standard error of the weight measurements.

Because the weight of the Foraminifera was close to the lower end of the measurement range, the accuracy of the measurements can be expected to be a function of weight, with presumably higher relative accuracy in heavier objects. To quantify this effect, we used specimens of *G. ruber* (white) and *G. elongatus* from the richest sample (cup number three; second half of March 2002, 42 specimens) to determine the relationship between individual standard errors (from repeated measurements of the same specimen) and the mean weight of that specimen. We found that lower weights indeed show higher relative standard errors (S1 File). While the relative standard error of the measurement is well below 4% for the majority of the shells, it can rise up to 8% for specimens lighter than 4 µg. Since nearly 75% of all weighed individuals are heavier than 4 µg, the resulting mean relative error is below 5%. On the basis of the weight (*W*_*i*_) and the cross-sectional area of the shell (*A*_*i*_), the individual area density (AD_*i*_) per specimen could be calculated as AD_*i*_ = *W*_*i*_/*A*_*i*_ [9].

Environmental data were retrieved from online repositories. Monthly sea surface temperature (SST) is based on the ICOADS 2° dataset provided by the NOAA/OAR/ESRL PSD (http://www.esrl.noaa.gov/psd/). Monthly sea surface salinity (SSS) data were taken from the EN4.0.2 dataset [52]. Weekly surface chlorophyll *a* concentrations of the surface water were retrieved from the U.S. Joint Ocean Flux Study [53]. All data were averaged for the approximated catchment area (31.65°–35.70° N, 19.51°–26.96° W [30]) of the sediment trap and the sampling interval of the respective sample. Data from the ESTOC series [54], which are available for several months of the investigated time interval [55], were used to independently verify estimates of the regional carbonate saturation using CO2Sys.

### Data Analysis

All statistical analyses of the data were conducted using the software R v. 3.1.0 [56]. Confidence intervals for sample means of shell size and calcification intensity were calculated by bootstrapping using the R-package “boot” v. 1.3-10 [57]. We used basic bootstrapping when the data showed a significant skewness, and accelerated bootstrapping when they did not [58]. Skewness was considered significant when the skewness (calculated according to equation I, table 1 in Tabor [59]) was larger than its approximated standard deviation. The normality of data distribution was tested with a Shapiro–Wilk test [60], while the homoscedasticity of data was tested with a Fligner–Killeen test [61].

To test the linearity of the size–weight regression within samples, the shell cross-sectional area was first scaled to volume by taking the area to the power of 3/2. The transformed size data were subjected to a Kendall–Theil robust line fitting [62–64], implemented in R on the basis of equations from Helsel and Hirsch [65], against the dependent variable shell weight. The slope (including its 95% confidence interval) of the resulting regression line and the strength of the relationship (coefficient of determination *R*^2^) were calculated. Since not all species were abundant enough in all samples to yield significant results, we only used the slopes of regressions that were significant at *α* = .05 in the ensuing analyses. To determine whether or not the relationship between size and weight is linear within the investigated size range, each linear regression model was tested against an exponential model using the *F*-distribution [66].

Differences in size–weight scaling among species were analyzed using a Kruskal–Wallis test [67], with ensuing pairwise Mann–Whitney *U* tests [68] (with *p*-values corrected for the false discovery rate [69]). To test for the influence of environmental parameters on the stability of the size–weight relationship, we used a robust multiple linear regression between the regression slopes against multiple candidate controlling variables on the basis of the MM-estimate [70], as implemented in the R-package “robust” v. 0.4-15. To test for the influence of environmental parameters on the calcification intensity of the shells of each species, we applied generalized linear models (GLM) [71] based on the gamma distribution, with identity as link function. A GLM analyzes the reaction in one dependent variable to several independent variables at the same time. For all GLMs we tested the significance of residual deviance, which tests if the model explains the data well, or if predicted values and residuals are correlated (which would indicate a biased analysis). GLMs of increasing complexity incorporate the potential influence of increasingly more environmental parameters, and thus necessarily explain a higher degree of the observed variation, reducing the amount of unexplained variation *ϵ*. The models were therefore ranked using the corrected Akaike information criterion (AIC_*c*_) [72] calculated with the R-package “bbmle” v. 1.0.16. The AIC_*c*_ can be used to infer, whether the increase in explanatory value of a more complex model is worth the higher complexity of that model, and the most parsimonious model was used for further interpretation.

## Results

### Fluxes of the Analyzed Species

**Fig 4:**
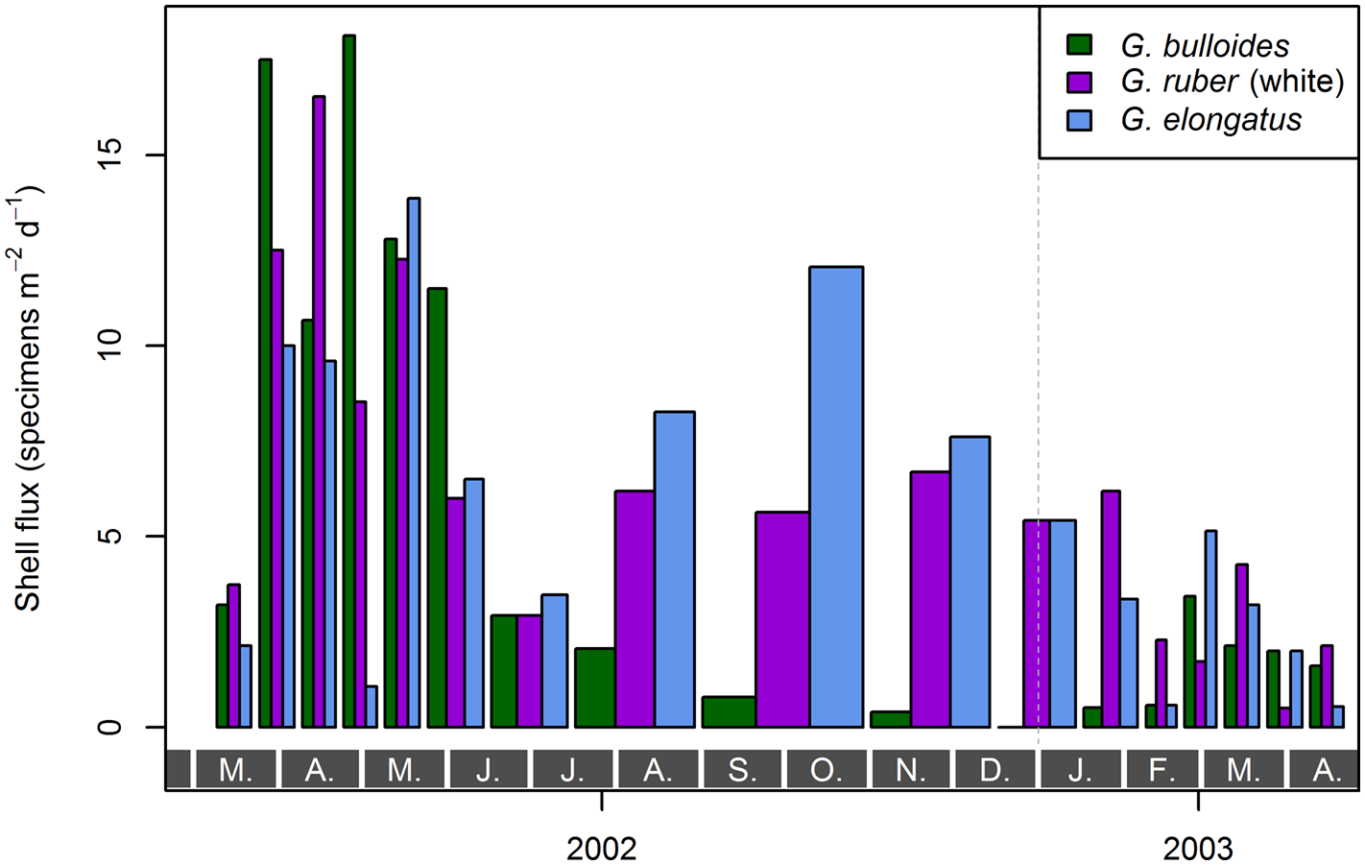
Flux of *Globigerina bulloides*, *Globigerinoides ruber* (white), and *Globigerinoides elongatus* sampled from March 2002 until April 2003 with sediment trap L1/K276. The flux was calculated on the basis of counted absolute abundances in the size fraction >150 µm, the trap opening of 0.5 m^2^, and the sampling duration of each sample. Sampling durations per cup differed, as is expressed by the varying width of the bars. Gray boxes with letters at the bottom indicate months, the vertical, dashed, gray line marks the end of 2002.

The shell fluxes in the fraction larger than 150 µm are shown in Fig 4. *Globigerina bulloides* showed lower mean flux (5 specimens m^−2^ d^−1^) than *G. ruber* (white) combined with *G. elongatus* (11 specimens m^−2^ d^−1^). The flux of *G. ruber* (white) and *G. elongatus* were on average rather similar.

Throughout the sampling period, all three species showed highest flux between March and May 2002, and generally lower flux during all other months, including March and April 2003; though *G. elongatus* displays a second peak in flux between August and September 2002 (Fig 4). From March to June 2002, *G. bulloides* generally showed the highest flux of all species, followed by *G. ruber* (white) and *G. elongatus*. For the rest of the investigated time interval the flux values were generally reversed, with *G. elongatus* mostly showing the highest flux before *G. ruber* (white) and *G. bulloides* (Fig 4).

### Shell Size

**Fig 5:**
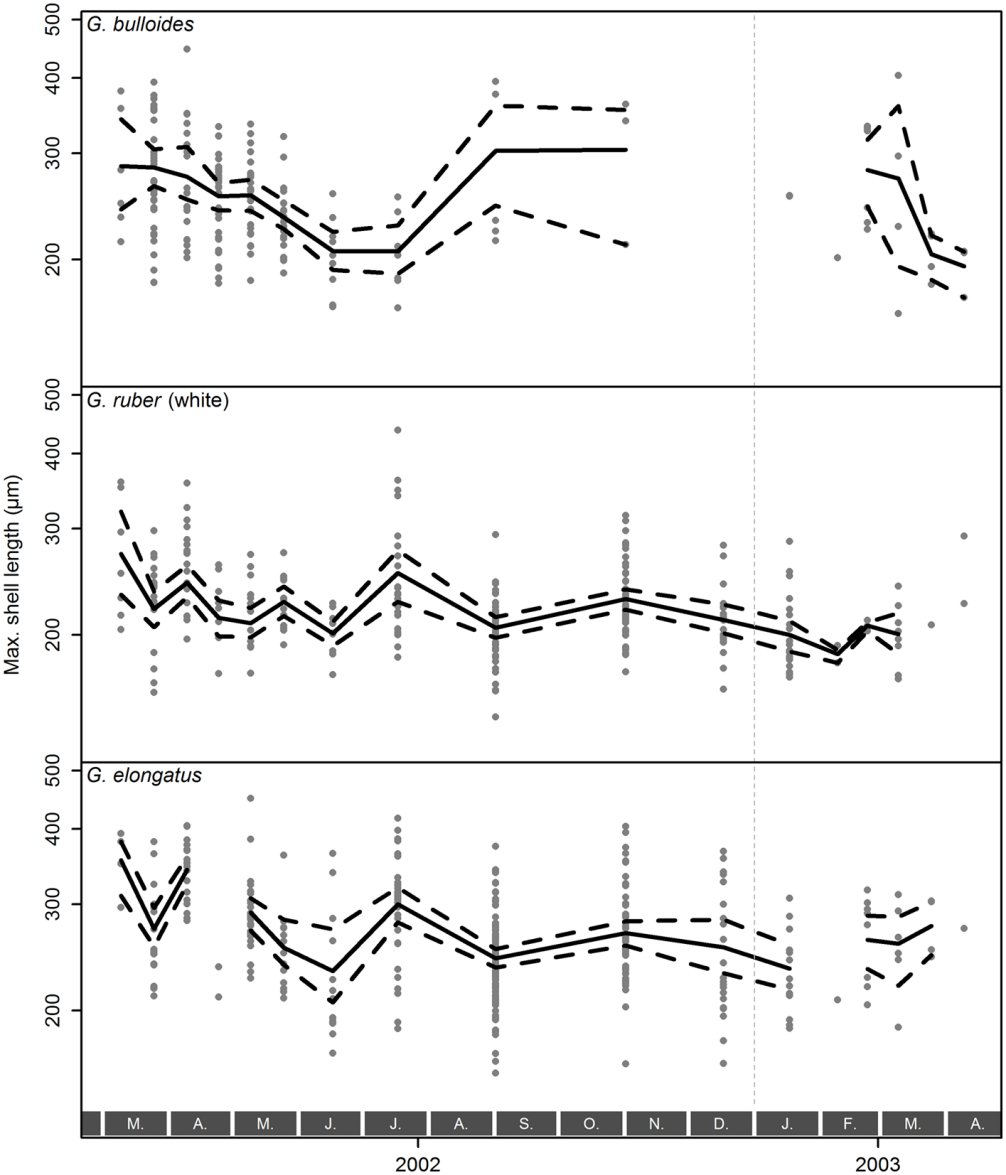
Shell sizes of *Globigerina bulloides*, *Globigerinoides ruber* (white), and *Globigerinoides elongatus* sampled from March 2002 until April 2003 with sediment trap L1/K276. Raw size values (expressed as Feret diameter) are indicated as symbols. The mean size per species per sample (solid lines) and its bootstrapped 95% confidence interval (dashed lines) is also shown. Gray boxes with letters at the bottom indicate months, the vertical, dashed, gray line marks the end of 2002. Note the log-scaling of the *y*-axis.

Shell sizes (Feret diameter, Fig 5) in *Globigerina bulloides* range between 162.7 µm and 446.4 µm (mean: 257.2 µm, median: 244.3 µm). Although on average the size distribution of both *Globigerinoides* species is similar, ranging from 146.2 µm to 449.3 µm (mean: 245.0 µm, median: 232.0 µm), *G. ruber* (white) (mean: 221.3 µm, median: 214.6 µm) is generally smaller than *G. elongatus* (mean: 267.0 µm, median: 260.4 µm). Shells of both *Globigerinoides* species are smallest during late winter and early spring and largest in March–April and around July. In contrast, *G. bulloides* shows smallest shell sizes in early to mid-summer (June–July) and relatively large shells during the rest of the year. In all species the shell size distribution is log-normal and unimodal in the vast majority of samples (S1 File), indicating the presence of only one statistical population.

### Shell Calcification

**Fig 6:**
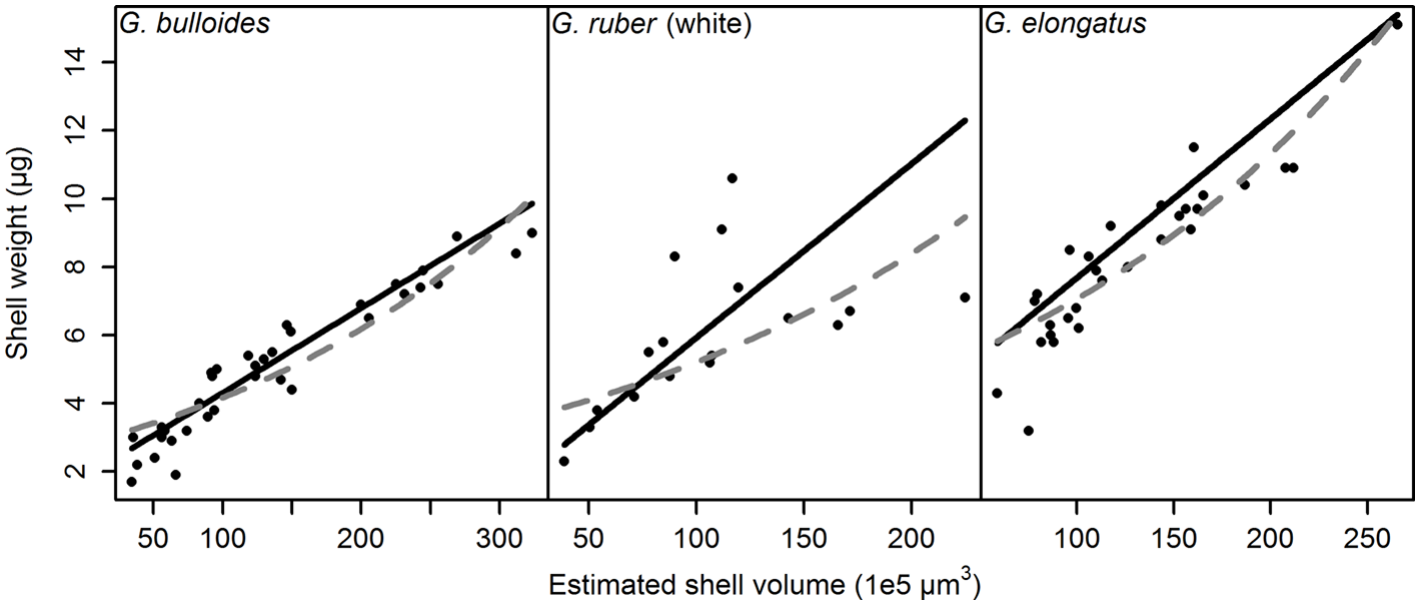
Calculation of the size–weight scaling per sample exemplarily shown in the richest sample each of *Globigerina bulloides* (Sample 3), *Globigerinoides ruber* (white) (Sample 4), and *Globigerinoides elongatus* (Sample 10), sampled from March 2002 until April 2003 with sediment trap L1/K276. The approximated shell volume was estimated from the cross-sectional area of the shell, and its relationship with shell weight was tested using a Kendall–Theil robust line fitting (solid black lines). The slope of the regression line corresponds to the size–weight scaling plotted in Fig 7. The best fitting exponential function through the points is also plotted as dashed gray line in each case, but in the example it would only in the case of *G. elongatus* significantly increase the fit of the model.

The relationship between size (scaled to volume) and weight of individual shells in representative samples is shown in Fig 6 (see also S1 File). In all samples and for all species, we observe strong linear relationships between the two variables, indicating a constant scaling between size and weight within the studied size range. To test this conclusion explicitly, we determined the exponential regression through the same points and checked for a significant increase in *R*^2^. The *R*^2^-value could be significantly increased in ca. 14% of the cases, but was decreased in nearly 35% of the cases by fitting an exponential function (S1 File), confirming that the scaling between size and weight is linear within the studied size range.

**Fig 7:**
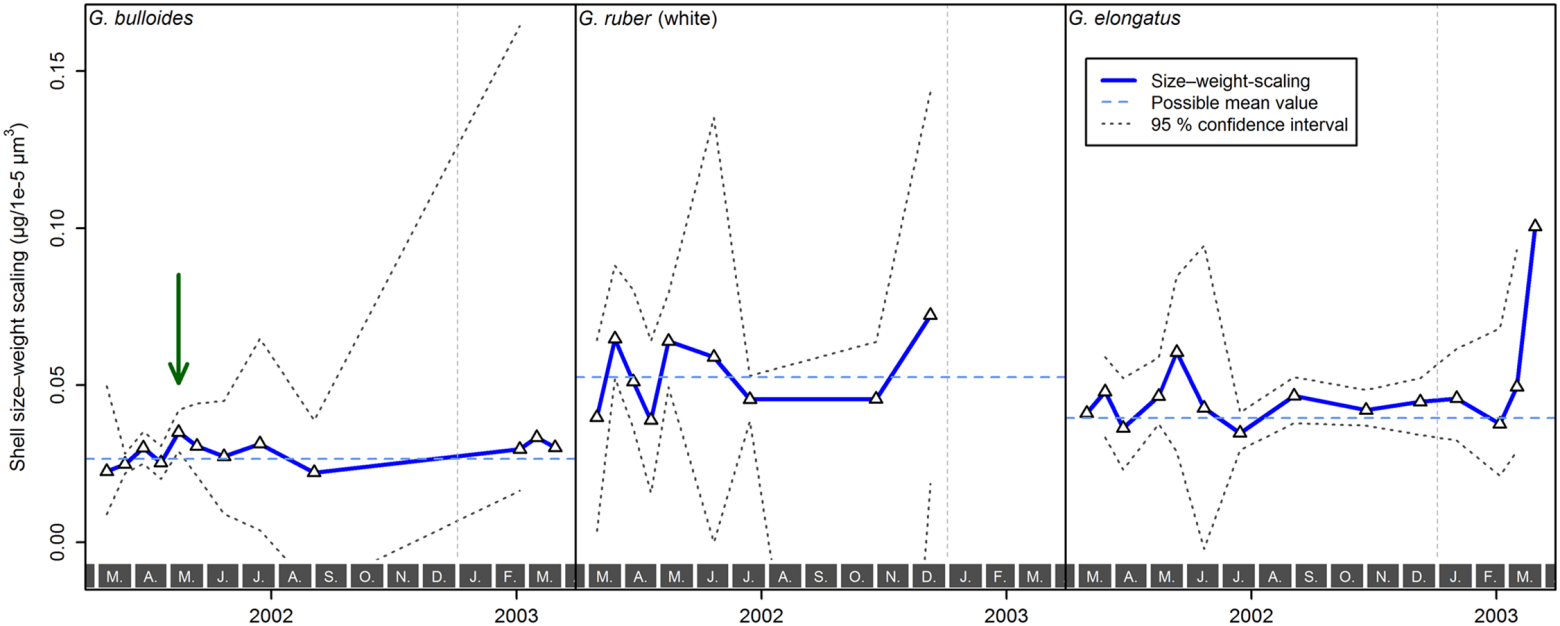
Estimated size–weight scaling and its 95% confidence interval of *Globigerina bulloides*, *Globigerinoides ruber* (white), and *Globigerinoides elongatus*, sampled from March 2002 until April 2003 with sediment trap L1/K276. Some samples either contained no or too few specimens to estimate the scaling or its confidence interval, or were not significant in the Kendall–Theil robust line fitting, and were thus not included in the analysis. A possible annual mean value of the size–weight scaling per species, that would never fall outside the 95% confidence interval, is indicated as well (in *Globigerina bulloides* this only works if one sample from early May 2002, marked by the green arrow, is regarded as an outlier). Gray boxes with letters at the bottom indicate months, the vertical, dashed, gray line marks the end of 2002.

**Fig 8:**
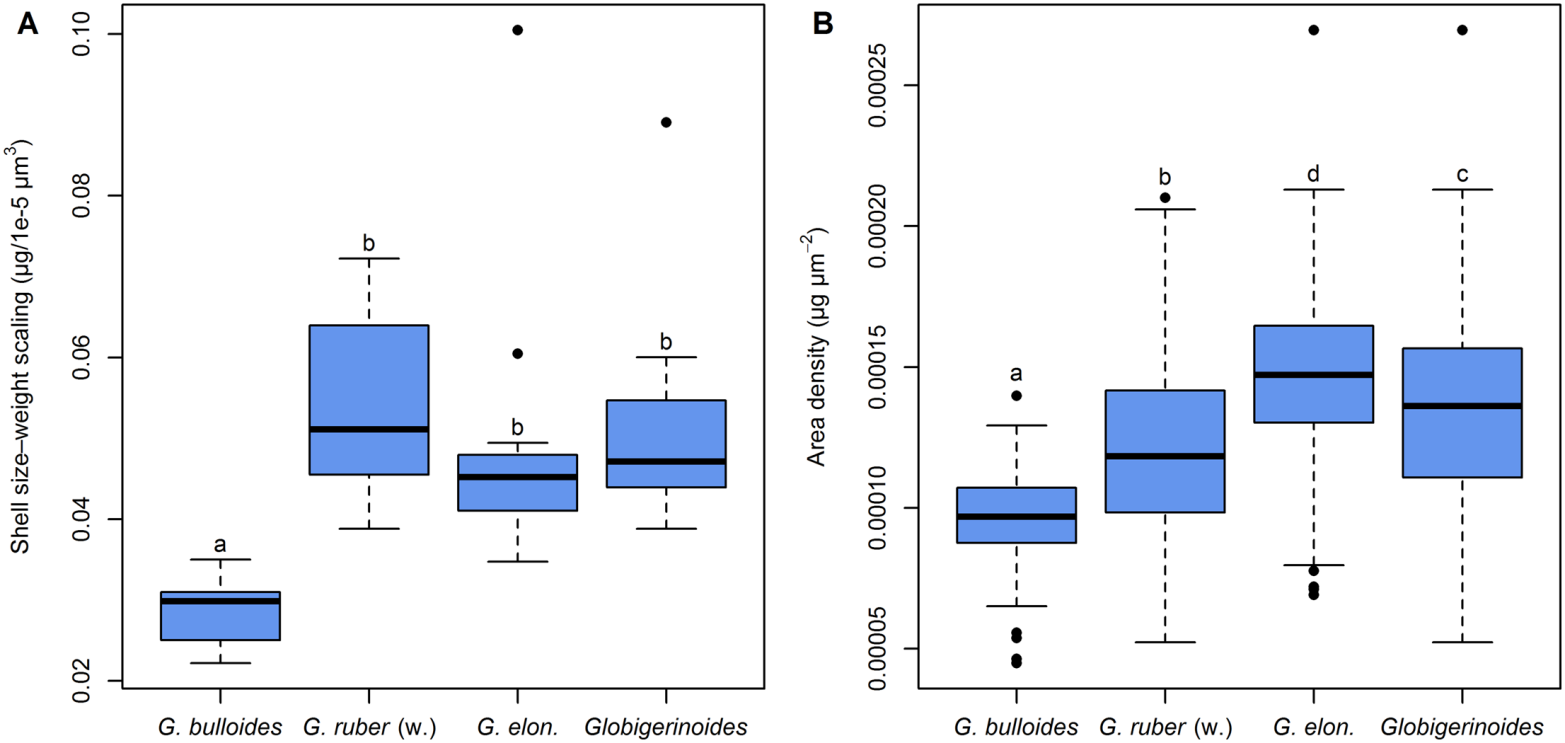
Size–weight scaling (A) and calcification intensity (B, expressed as area density) of *Globigerinoides ruber* (white), *Globigerinoides elongatus*, both *Globigerinoides* species pooled together, and *Globigerina bulloides* sampled from March 2002 until April 2003 with sediment trap L1/K276. Boxplots show the median (thick line), interquartile range (blue box), 1.5× interquartile range (whiskers), and outliers (black dots). The group assignment according to pairwise comparisons (compare Tables 1 and 2) is indicated by lower case letters above the whiskers, where groups that do not differ significantly from each other are marked with the same letter.

Having established that the slope of a linear regression can be used to describe the size–weight relationships within samples, we can investigate differences among samples and species. First, we note that variation within species is smaller than differences among species (Fig 8A). The size–weight slope of *G. bulloides* appears consistently much smaller than that of *G. ruber* (white) and *G. elongatus*. The slope values of *G. ruber* (white), *G. elongatus*, and the pooled *Globigerinoides* species were statistically indistinguishable (Table 1). Second, we examine the temporal evolution of the size–weight slope values for all three species (Fig 7). This plot reveals that only in one *G. bulloides* sample does the slope deviate significantly from the average value for the species. The significance is here assessed by finding a hypothetical value of the size–weight slope that falls within the 95% confidence interval of as many samples as possible. Because the majority of the slope values for each species do not deviate significantly from each other, it seems that the size–weight scaling within each species did not change throughout the studied period.

**Table 1:** Pairwise comparison of the size–weight scaling slope.

Species 1	Species 2	adj. *p*-value
*Globigerinoides*	*G. bulloides*	<.001
*Globigerinoides*	*G. ruber* (white)	.493
*Globigerinoides*	*G. elongatus*	.348
*G. ruber* (white)	*G. elongatus*	.337
*G. ruber* (white)	*G. bulloides*	<.001
*G. elongatus*	*G. bulloides*	<.001

Comparison between *Globigerinoides ruber* (white), *Globigerinoides elongatus*, both species of *Globigerinoides* pooled together, and *Globigerina bulloides* sampled from March 2002 until April 2003 with sediment trap L1/K276, based on a Mann–Whitney *U* test with *p*-values adjusted for the false discovery rate. *Globigerina bulloides* shows a significantly different size–weight scaling than *Globigerinoides*, but the scaling of *G. ruber* (white) and *G. elongatus* are indistinguishable (compare Fig 8A).

**Fig 9:**
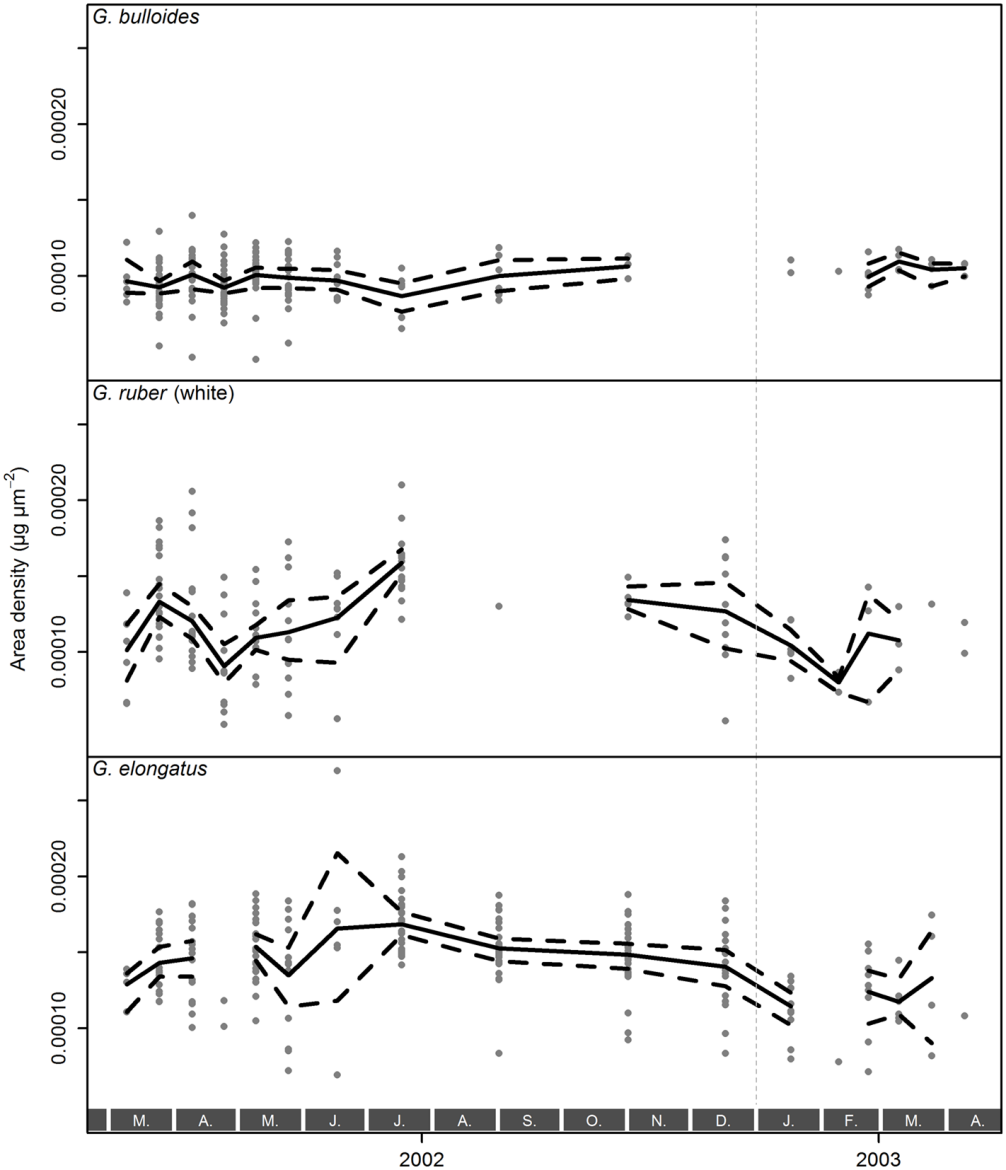
Calcification intensities (expressed as area density) of shells of *Globigerina bulloides*, *Globigerinoides ruber* (white), and *Globigerinoides elongatus* sampled from March 2002 until April 2003 with sediment trap L1/K276. Raw values (gray dots) are plotted together with the mean value (solid lines) and its bootstrapped 95% confidence interval (dashed lines) per sample. Gray boxes with letters at the bottom indicate months, the vertical, dashed, gray line marks the end of 2002.

Because the size–weight scaling seems constant through time for each species and the distribution of AD_*i*_ values within nearly all samples is unimodal (S1 File), it is possible to use the average AD of all specimens within a sample as a robust estimate of calcification intensity. This is equivalent to comparing the intercepts of the size–weight regression lines, assuming their slopes are the same. The resulting values represent a reliable form of size-normalized weight. The temporal evolution of calcification intensity in all three species is shown in Fig 9. This reveals that calcification intensity of *G. ruber* (white) and especially *G. elongatus* seems to be lower during late winter and the highest values are reached during June and July. In contrast, calcification intensity of *G. bulloides* appears to be rather constant throughout the year. In all samples, the calcification intensity of *G. elongatus* is generally larger than that of *G. ruber* (white) and the values for *G. bulloides* are consistently the smallest (Fig 8B, Table 2).

**Table 2:** Pairwise comparison of the calcification intensity.

Species 1	Species 2	adj. *p*-value
*Globigerinoides*	*G. bulloides*	<.001
*Globigerinoides*	*G. ruber* (white)	<.001
*Globigerinoides*	*G. elongatus*	<.001
*G. ruber* (white)	*G. elongatus*	<.001
*G. ruber* (white)	*G. bulloides*	<.001
*G. elongatus*	*G. bulloides*	<.001

Comparison between *Globigerinoides ruber* (white), *Globigerinoides elongatus*, both species of *Globigerinoides* pooled together, and *Globigerina bulloides* sampled from March 2002 until April 2003 with sediment trap L1/K276, based on a Mann–Whitney *U* test with *p*-values adjusted for the false discovery rate. The calcification intensity (expressed as area density) is different between all species, and most importantly the calcification intensity of the pooled *Globigerinoides* is not representative for either of the two species pooled together (compare Fig 8B).

## Discussion

### Scaling of Size and Weight among Individual Shells

All methods used to quantify calcification intensity in planktonic Foraminifera normalize weight to shell size, assuming that the scaling of size and weight is consistent within the analyzed size range. Alternatively, if the scaling between size and weight varied across communities, differences in calcification intensity determined in a given size fraction could exist for two reasons (Fig 1). First, an observed offset could reflect an offset in the scaling line, indicating that the shells in one of the populations were more heavily calcified irrespective of size. Second, the same difference could reflect a change in the slope of the scaling line, implying that in one of the populations compared to the other, larger shells were more heavily calcified than smaller shells. These alternatives would imply fundamentally different processes responsible for the same amount of observed change in calcification intensity, when expressed as some form of size-normalized weight.

Theoretically, because the analyzed planktonic Foraminifera belong to the same spinose clade, have the same general shell morphology, and build their shells in a similar way, there should be no *a priori* reason why the scaling slopes between shell size and weight should be different. In this way, the assumption of the classical methods to quantify calcification intensity appears justified. Indeed our analysis reveals that the scaling, when expressed as volume to weight relationship, is consistent within each species and does not change for populations exposed to the contrasting summer or winter conditions (Fig 7). However, we observe statistically significant, consistently large differences in the scaling slope between *Globigerina bulloides* and both *Globigerinoides* species (Fig 8A). This means that there is not a universal scaling slope between size and weight among planktonic Foraminifera. If we consider that all planktonic Foraminifera commence calcification as geometrically similar prolocular stages [73], then different size–weight scaling slopes among species in their adult stage must result from different ontogenetic trajectories in this scaling. The differences in scaling imply that absolute values of the size-normalized weight (irrespective of its precise formulation) are not comparable among species. Although we only observe differences in the scaling slope between species, it cannot be excluded that such differences also occur within species [25].

**Fig 10:**
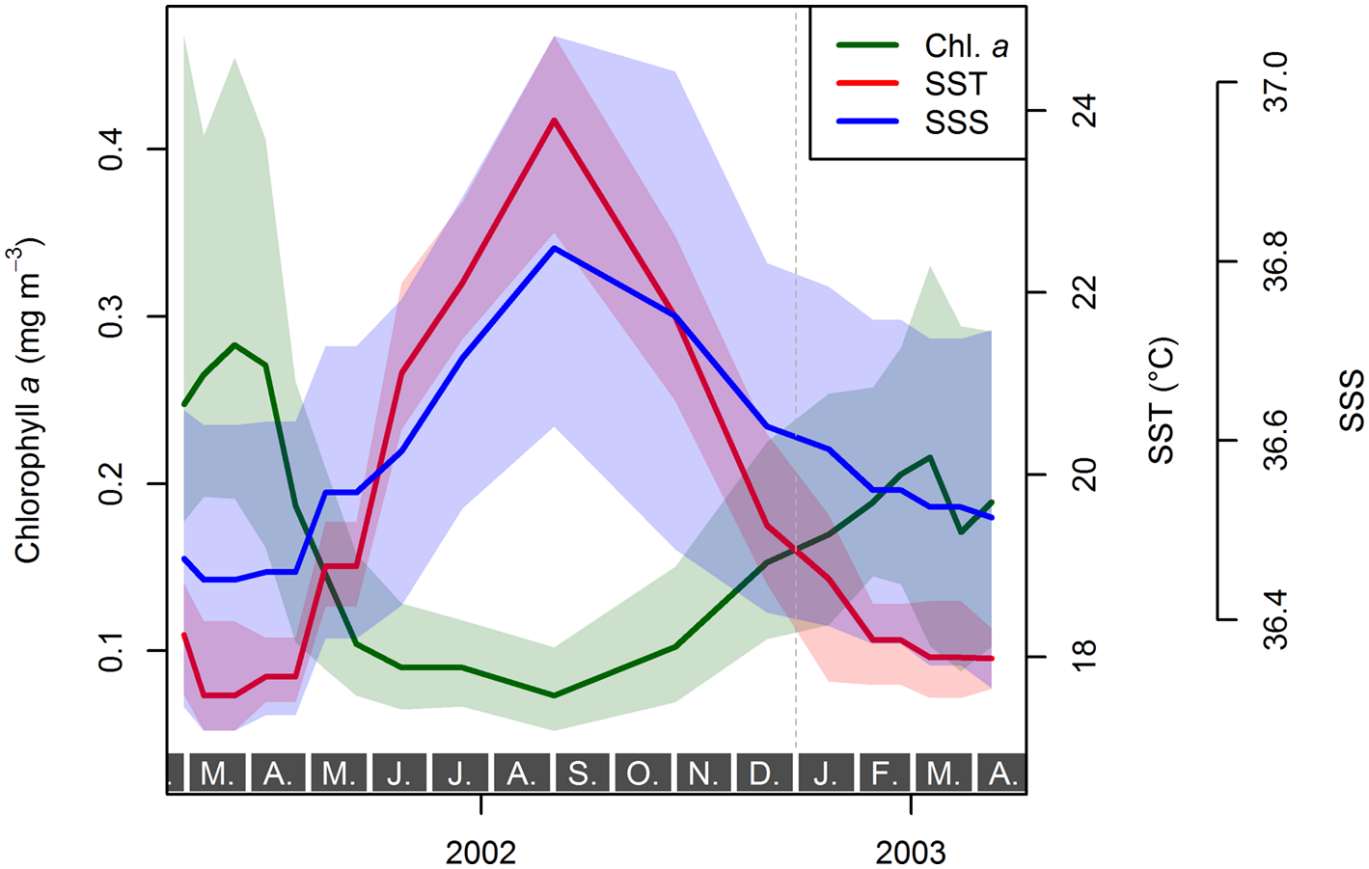
Mean values (lines) and range (shaded area) of chlorophyll *a* content of the surface waters [53], sea surface temperature (SST, ICOADS data provided by the NOAA/OAR/ESRL PSD, http://www.esrl.noaa.gov/psd/), and sea surface salinity (SSS, EN4.0.2 dataset [52]) for the sampling period in the catchment area of sediment trap L1/K276. Since salinity is highly correlated with temperature and affects the carbonate saturation state only by a factor of ten less than temperature, we did not include it in our analyses for the environmental forcing of shell calcification.

The lack of significant temporal variation in the scaling slope between size and weight within individual species may reflect the large confidence intervals on the slope, which are mainly a function of sample size. Therefore, we investigated whether the observed variation in slope within species correlates with any of the candidate environmental parameters: temperature, as main factor influencing the pace of cellular processes, and chlorophyll *a* concentration as an indicator of productivity and nutrient availability (Fig 10). Given that the calcification intensity of foraminiferal shells is itself considered to be driven by one or more of those environmental parameters, a correlation of the size–weight scaling with the same environmental parameters would introduce a cross-correlation that would render the calcification intensity prone to misinterpretation. A robust multiple linear regression of the scaling slope values indicates no significant effect of either environmental parameter in any of the species investigated (Table 3), however. While this does not rule out the environmental forcing of the scaling slope with any as yet untested environmental factor, this analysis seems to support the observation that the scaling slope may be an intrinsic characteristic of each species, which is invariant to environmental perturbations. If this conclusion holds, then size-normalized weight can be used as a proxy for calcification intensity within species.

**Table 3:** Robust multiple linear regression *p*-values for the influence of environmental parameters on the size–weight scaling.

	*G. bulloides*	*G. ruber* (white)	*G. elongatus*	*Globigerinoides*
Intercept	.022	.934	.023	.730
Chlorophyll *a*	.156	.967	.073	.836
SST	.144	.970	.141	.860

Data for *Globigerinoides ruber* (white) and *Globigerinoides elongatus*, both *Globigerinoides* species combined, and *Globigerina bulloides* sampled from March 2002 until April 2003 with sediment trap L1/K276. Neither chlorophyll *a* content nor temperature (SST) had an influence on the scaling slopes in any species.

### Measuring Calcification Intensity

Our analyses of the size–weight scaling have shown that calcification intensity within species can be approximated by size-normalized weight, at least as long as only a narrow size fraction is used for such studies. Because of the availability of single-shell measurements, we can here use and analyze the distribution of area density values as applied by Marshall et al. [9]. This approach is exact, but time-consuming. Therefore we test how the mean AD values would differ from a mean area density (MAD) determined such that the size of the specimens is measured individually, but all specimens from a single sample are weighed together [20]. Confidence intervals for the MAD can be estimated by repeated determination of the MAD in random subsamples of one sample, and bootstrapping confidence intervals for variable sample sizes on the basis of that dataset [20]. We simulated MAD values for all three species and compared them with the mean of the individual AD_*i*_ values per sample (S1 File). We found that both values are highly and significantly correlated, with the slope of the regression line in no case being significantly different from one. We can thus conclude that the more efficient method of MAD is likely to yield comparative results to the more time-consuming determination of individual AD_*i*_ values. However, when comparing the bootstrapped confidence interval for MAD calculated as in Weinkauf et al. [20] with the confidence interval determined from the distribution of the individual AD_*i*_ values, a significant mismatch is observed. The bootstrap procedure tends to underestimate the uncertainty of the MAD values on average by approximately a factor of two (S1 File). Thus, whilst the MAD approach seems to yield reliable mean values, the associated uncertainty seems difficult to estimate. As shown previously, both AD and MAD methods are likely to be much superior to the sieve-based approaches, especially to the unqualified sieve-based weight [51].

An interpretation of calcification intensity measured in this way requires that AD and MAD are independent of the size–weight scaling. While there is no statistically significant systematic change of this scaling in the course of a year or in relation to the environment, there remains a certain variation within species that appears unexplained (compare Figs 7 and 8). Should this variation be systematically linked to calcification intensity, then the observed calcification intensity could at least partly reflect changes in the size–weight scaling, and the resulting difference in calcification intensity would be difficult to interpret. To exclude this possibility, we tested for such a relationship in all three species by applying a Kendall–Theil robust line fitting to the calcification intensity in dependence of the size–weight scaling slope. The relationship was not significant in any of the three species (*p* >.238, S1 File), indicating independence of the variability in both values and supporting the interpretation that the observed variation in the size–weight scaling slope within species is stochastic.

A further prerequisite for the validity of such an analysis is that the shell calcification has not been inconsistently changed within the assemblage, as it could appear for instance by secondary calcite dissolution during the settling of the foraminiferal shells or within the sample cups over time due to organic degradation. As far as the calcite dissolution during settling of the shells to the sediment trap depth of 2000 m is concerned, we note that the foraminiferal shells do not show any signs of calcite dissolution on their surfaces. This is confirmed by the fact that other studies have successfully used sediment trap samples from comparable depths for calcification studies without ever noting any related problems with calcite dissolution in the water column [8, 17]. Even if calcite dissolution during the settling process would play a significant role, this could be ignored for this study. Since all used species live in comparable depths and have a comparable morphology, they would all be equally influenced by such a process, so that in the worst case the absolute values we obtained, but not their trends, could have been corrupted. To prevent calcite dissolution within the cups due to organic degradation, they were buffered with sodium acide [30]. This procedure seems to have been very successful in preventing calcite dissolution, since Storz et al. [39] reported consistently low foraminiferal dissolution on the basis of shell fragmentation, and we also found aragonitic pteropods in the sample material. Furthermore, should calcite dissolution within the sample cups play an important role, we would expect a cumulative impact over time, that would lead to consistently lower apparent calcification intensities of Foraminifera from older cups that have been exposed to degrading conditions for a longer timespan. Conversely, no such trend is visible in our data (Fig 9).

### Determinants of Calcification Intensity within Species

Considering the previous analyses, we may now use the temporal development of calcification intensity per species to search for potential controlling environmental parameters. This analysis is to some degree complicated by the fact, that the environment is not fully consistent within the catchment area of the sediment trap [30], and thus the investigated samples are a mixture of individuals exposed to slightly varying environments. However, Fig 10 clearly shows that the annual variation is consistently much larger than the range of the environmental parameters within the catchment area at any given time. Furthermore, van Sebille et al. [74] globally quantified drift distances and related temperature offsets of planktonic Foraminifera during their lifetime. They imply that the sampling region is consistently characterized by an average drifting distance of only ca. 30–60 km which is associated with temperature offsets in regard to the place of sedimentation of the shell of close to 0°C. We thus conclude that the results of our analysis are not biased by any significant or systematic analytical problems introduced by the spatial variation of the environment.

We have chosen this study area because it is characterized by a very stable carbonate system. Using long-term observations available for the catchment area, carbonate saturation is supposed to change by only 7 µmol kg^−1^ during the year, with the large majority of change occurring in autumn. Coeval *in situ* measurements from the ESTOC series [54] support our assumption of a stable carbonate system, with highest [CO_3_^2−^] of <240 µmol kg^−1^ in autumn and a maximum variation of 18 µmol kg^−1^ per year. Disregarding the autumn months, which did not show large deviations in calcification intensity in any species (Fig 9), the yearly variation in [CO_3_^2−^] is less than 8 µmol kg^−1^. Therefore, the explanation of the observed variation in calcification intensity has to consider the remaining candidate parameters: temperature and chlorophyll *a* concentration. We disregard salinity because it is highly correlated with temperature (Fig 10) and could only influence calcification by influencing the carbonate system, which is only to a minor degree driven by salinity. To this end, we fitted a series of GLMs.

The greatest problem in such an analysis is the often occurring multicollinearity between environmental parameters. To that end, we performed a Pearson product-moment correlation between temperature and productivity. Considering a significant correlation with a correlation coefficient of >0.7 as a sign for significant collinearity [75], we observe significant collinearity between temperature and chlorophyll *a* (*r* = −0.828, *p* <.001). However, because of the fact that high collinearities in a GLM increase the chance of a type II error, thus making it less likely to detect a significant relationship, without increasing the false-positive rate [75], we conclude that we can perform the intended analyses on our data, but realize that the statistical tests are likely to be too conservative. The residual deviance was insignificant (*p* = 1) for all our analyses, indicating an unbiased analysis with a high explanatory value of the chosen models.

We begin with an analysis of the genus *Globigerinoides*. The most simple Model (1) pools both species of *Globigerinoides* and includes as candidate controlling variables the SST (*T*) and chlorophyll *a* content (*Ca*). While *Globigerinoides* (as a symbiont bearing genus) may by itself be less dependent on productivity, favorable conditions for the phytoplankton might also indicate favorable conditions for the foraminiferal symbionts, which is why productivity has been considered in the model. Some authors suggested that calcification intensity of foraminiferal shells reflects growth under optimal environmental conditions [16, 17, 76, 77]. Assuming that the suitability of an environment directly influences the abundance of the species, a more complex Model (2) thus assumes that the AD also changes as a function of the flux (*F*) of the taxon. Next, Model (3) assumes that the different though closely related species (*Bs*) *G. ruber* (white) and *G. elongatus* may calcify differently under all conditions. Finally, Model (4) expands this hypothesis by assuming that different species may also show different reactions in their shell calcification towards changes in the environmental parameters.

$$\begin{array}{c} \text{AD}=x_{1}\;T+x_{2}\;Ca+\epsilon \end{array}$$ (1)

$$\begin{array}{c} \text{AD}=x_{1}\;T+x_{2}\;Ca+x_{3}\;F+\epsilon \end{array}$$ (2)

$$\begin{array}{c} \text{AD}=x_{1}\;T+x_{2}\;Ca+x_{3}\;F+x_{4}\;Bs+\epsilon \end{array}$$ (3)

$$\begin{array}{ccc} \text{AD} & = & x_{1}\;T+x_{2}\;Ca+x_{3}\;F+x_{4}\;Bs+x_{4}\;Bs\times x_{1}\;T\\ & & +\;x_{4}\;Bs\times x_{2}\;Ca+x_{4}\;Bs\times x_{3}\;F+\epsilon \end{array}$$ (4)

The analysis shows that Model (4) explains the data best. Following the Akaike information criterion test, Model (3) (ΔAIC_*c*_ = 16.5), Model (2) (ΔAIC_*c*_ = 57.2), and Model (1) (ΔAIC_*c*_ = 57.6) were all clearly inferior.

**Fig 11:**
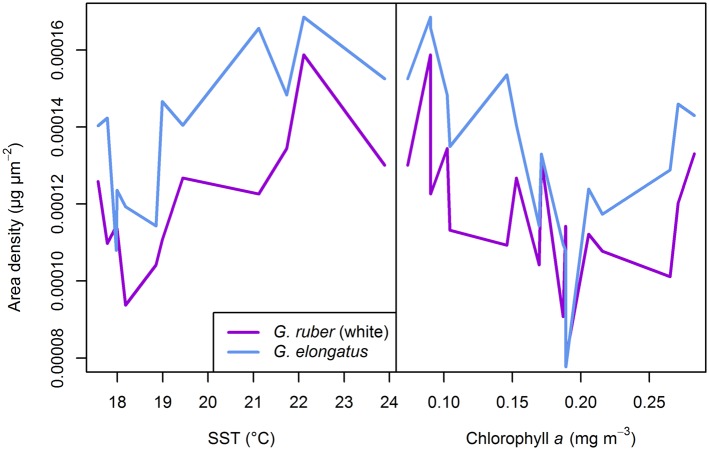
Interaction plot of the calcification intensity in *Globigerinoides ruber* (white) and *Globigerinoides elongatus* sampled from March 2002 until April 2003 with sediment trap L1/K276. While both species generally show a positive correlation of area density with sea surface temperature (SST) and a negative correlation with surface water productivity, the variable offset between the lines shows that both species differ in base calcification intensity and react differently to changes in the environment (compare Table 4).

An examination of the coefficients of Model (4) allows an assessment of factors that most influence the calcification intensity within the two *Globigerinoides* species. This reveals that the pooled *Globigerinoides* calcification is mainly driven by SST and chlorophyll *a*, with higher calcification intensities observed during times of raised SST and lower productivity (Fig 11, Table 4). Both species show a constant offset in calcification intensity, confirming results already discussed above, as well as a significant interaction term between species, and SST and chlorophyll *a*, implying that calcification in *G. ruber* (white) and *G. elongatus* respond differently to changes in temperature and productivity. Shell flux (as indicator of the suitability of the environment) does not seem to affect shell calcification in the genus, but the interaction term between species and flux is significant. When applying a Kendall–Theil robust line fitting solely on the correlation between flux and AD (i.e. disregarding other factors) one indeed finds a significant positive correlation (*p* <.001, *R*^2^ = 0.459) between abundance and shell calcification in *G. elongatus*, but not in *G. ruber* (white) (*p* <.564, *R*^2^ = −0.006) (S1 File). We must therefore conclude that calcification intensity is indeed influenced by the suitability of the environment in some but not all species.

**Table 4:** Results of the most informative generalized linear model (Model 4) for the calcification intensity of *Globigerinoides* species from trap L1/K276 in the North Atlantic.

	Standard error	*t*-value	*p*-value
Intercept	5.169 × 10^−5^	−5.045	<.001
SST	2.381 × 10^−6^	7.375	<.001
Chl. *a*	5.213 × 10^−5^	4.584	<.001
Shell flux	4.955 × 10^−7^	0.896	.370
Biospecies	7.127 × 10^−5^	4.522	<.001
SST × biospecies	3.201 × 10^−6^	−4.465	<.001
Chl. *a* × biospecies	7.940 × 10^−5^	−3.059	.002
Shell flux × biospecies	8.278 × 10^−7^	2.197	.029

The model implies that calcification is mainly driven by temperature and surface water productivity, but not by environmental suitability as indicated by shell flux. It further confirms the results of the Mann–Whitney *U* test that the two species *G. ruber* (white) and *G. elongatus* differ in base calcification intensity (compare Fig 8B, Table 2), but further implies that they also show different reaction terms to virtually all environmental parameters.

Until now, there has been little evidence for an influence of temperature on calcification intensity. Only Marshall et al. [9] presented a significant relationship between area density and SST, but they attributed it to a collinearity of SST with [CO_3_^2−^], favoring carbonate chemistry as the main explanatory variable. Raised temperature leads to higher metabolic rates as well as higher carbonate saturation, thus favoring calcification both abiotically and biotically, providing excess energy for biomineralization. The latter only applies as long as the temperature increase does not exceed the physiological optimum of a species, however. The carbonate system has been rather stable during the time interval investigated, and largest changes in [CO_3_^2−^] concentration occurred in autumn, while the calcification intensity of shells of both *Globigerinoides* species showed largest trends during spring and summer (Fig 9). The observed variation in calcification intensity is thus well correlated with changes in SST and chlorophyll *a* content (Fig 10), when at the same time [CO_3_^2−^] varied by only ca. 1 µmol kg^−1^. Our data thus imply that the biotic component may play a major role in shell calcification intensity, regardless of changes in [CO_3_^2−^].

The evidence for a relationship between calcification intensity and optimum growth conditions in *G. elongatus* seems to be in support of an existence of energy trade-offs between calcification and growth under suboptimum conditions, as postulated before [12, 16]. On the other hand, the observed negative relationship of calcification intensity with productivity contradicts the idea that higher nutrient content of the surface water might indicate favorable conditions for the symbionts or that food availability alone may free more energy for calcification. Instead, we hypothesize that higher surface water productivity may lead to more light attenuation, so that under bloom conditions the foraminiferal symbionts receive less light, and calcification intensity is reduced, as observed in laboratory experiments manipulating the light levels in the symbiont-bearing species *Trilobatus sacculifer* (Brady, 1877) [78].

The discovery of different base calcification intensities and different reactions of calcification intensity to environmental variables between the two closely related species *G. ruber* (white) and *G. elongatus* is most interesting. Merging these species in analyses of calcification intensity would introduce an interaction term between species and their changing abundance, and the environment that cannot be controlled. As we will discuss below, patterns of calcification observed in past studies potentially lumping these forms under the same category may be severely affected by this interaction.

The general validity of the inferences based on the analyses of calcification intensity in *Globigerinoides* can be assessed by replicating the same analytical framework for calcification intensity data based on the species *G. bulloides*. For this species, only Models (1) and (2) can be considered because all data are derived from the same morphospecies. Replicating the analysis as carried out for *Globigerinoides*, we find for *G. bulloides* Model (2) to be the most informative, with Model (1) being clearly distinguishable and inferior (ΔAIC_*c*_ = 5.3). The model indicates that there is no influence of either SST or chlorophyll *a* on calcification intensity in this species (Table 5). The lack of reaction of shell calcification in *G. bulloides* towards SST could indicate that the temperature effect observed in *Globigerinoides* may be mediated by its symbionts. This would also be consistent with *G. bulloides* lack of reaction to chlorophyll *a* concentration.

**Table 5:** Results of the most informative generalized linear model (Model 2) for the calcification intensity of *Globigerina bulloides* from trap L1/K276 in the North Atlantic.

	Standard error	*t*-value	*p*-value
Intercept	2.814 × 10^−5^	5.339	<.001
SST	1.261 × 10^−6^	−1.772	.078
Chl. *a*	2.312 × 10^−5^	−1.015	.311
Shell flux	2.274 × 10^−7^	−2.788	.006

The model implies that calcification intensity in that species is influenced by environmental suitability, but is otherwise robust against environmental change.

**Fig 12:**
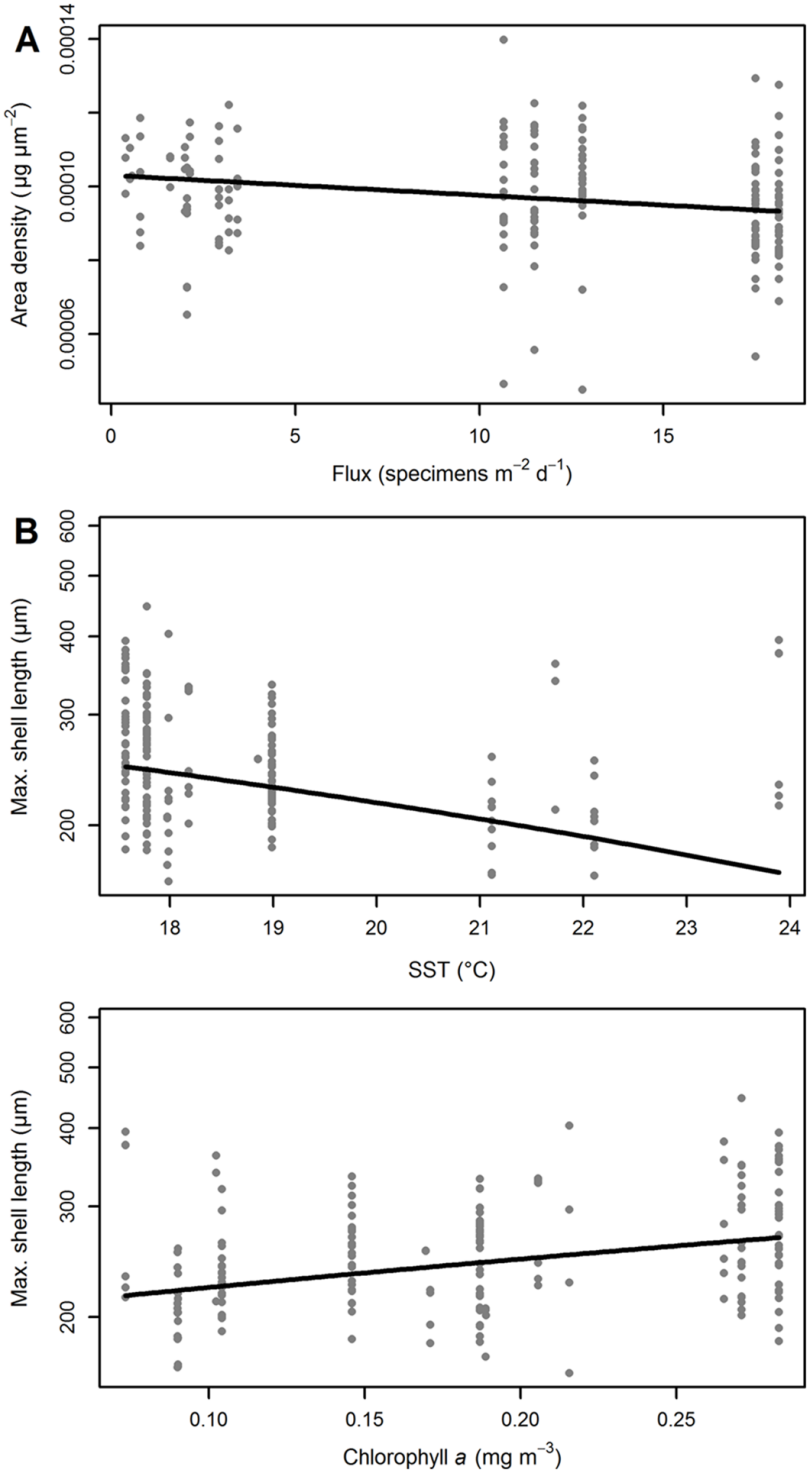
Cross plot of the calcification intensity (expressed as area density, AD) and shell size of *Globigerina bulloides* sampled from March 2002 until April 2003 with sediment trap L1/K276. Raw data values (gray dots) are plotted alongside the regression line from a Kendall–Theil robust line fitting. (A) The AD of shells of *G. bulloides* is decreasing when the species becomes more abundant (compare Table 5). (B) The shell size (Feret diameter) in *G. bulloides* shows a negative correlation with sea surface temperature (SST), but increases with productivity, likely due to the larger availability of nutrients and thus energy for the metabolism (compare Table 6). Note the log-scaling of the *y*-axis.

Model (2) further indicates a significant correlation between flux and calcification in *G. bulloides* (Fig 12A), which is also supported by a linear regression (compare S1 File). In contrast to predictions by de Villiers [16] and Naik et al. [17], however, this correlation is negative, i.e. higher abundances in *G. bulloides* are correlated with less calcified shells. This puzzling observation can be explained in two ways. First, if high abundance in the species reflects faster growth (i.e. more frequent addition of chambers), there is less time available for biomineralization and as a result the shells are less calcified than in less suitable environments. Alternatively, *G. bulloides* is known to harbor significant genetic diversity and the studied region is likely inhabited by as many as three different genetic lineages within the morphospecies [46]. If, like in *Globigerinoides*, these cryptic lineages have a species-specific response of calcification intensity to environmental parameters, the resulting “pooled” signal could be entirely confounded by this effect.

**Table 6:** Results of the generalized linear Models (1) and (2) for the shell size of *Globigerina bulloides* from trap L1/K276 in the North Atlantic.

	Standard error	*t*-value	*p*-value
Model (1)			
Intercept	81.842	0.475	.635
SST	3.726	2.130	.035
Chl. *a*	82.530	4.533	<.001
Model (2)			
Intercept	97.013	−0.570	.569
SST	4.354	2.750	.007
Chl. *a*	83.202	4.719	<.001
Shell flux	0.762	1.788	.075

The models show an influence of both sea surface temperature (SST) and productivity on the observed shell size.

### Species-Specific Calcification Patterns

Our results indicate that the response of calcification intensity to environmental parameters is species-specific. This applies not only to the existence of significant relations, but also to its sign. In addition, we have for the first time provided evidence that not only the calcification intensity, but also the size–weight scaling differs among species (Fig 8A). Although studies on the determinants of calcification intensity in planktonic Foraminifera have been conducted using different material and different methodology, there now exist enough data to attempt a comparison of the types of responses implied by individual studies (Table 7). Beside the fact that this study is one of the few investigating the effects of productivity on calcification, our results are broadly coherent with the patterns documented in earlier studies. The negative correlation between flux and calcification intensity in *G. bulloides* has been also observed by Aldridge et al. [15] in plankton samples from the North Atlantic. The fact that this counter-intuitive result has now been replicated may indicate that it hints at the existence of a poorly understood aspect of the environmental control of calcification in that species. It may still be an effect of combining different genetic lineages of this species in the analysis, but we note that in our study and that by Aldridge et al. [15], the genetic lineages that may have been pooled are likely to be different.

**Table 7:** Review of environmental parameters that have been found to influence foraminiferal shell calcification in several studies thus far, including the species and type of material used.

Symbionts	Species	Publication	Material type	Temperature	Temperature range (°C)	Productivity	Carbonate ion	[CO_3_^2−^] range (µmol kg^−1^)	Optimal growth conditions	other
+	*G. ruber* (d’Orbigny, 1839) ^d^	Davis et al. [14]	Fossil sediment	0	max. 7					
+	*G. ruber* (white)^c^	Gonzalez-Mora et al. [76]	Fossil sediment	+^a^	9		+*^a^	NA	0^a^	
		Beer et al. [12]	Plankton net samples				−	60	0	
		Naik et al. [77]	Fossil sediment	0	4		0*	NA	+	
		Mohan et al. [21]	Plankton net samples and surface sediment	+^a^	NA					
+	*G. ruber* (white) + *G. elongatus*	de Moel et al. [7]	Surface sediment				+*	max. 18		influence of monsoon seasonality
		Naik et al. [17]	Trap samples	0	max. 4		0*	NA	+	
+	*G. ruber* (white)	This study	Trap samples	+	7	−	0*	7	0	
+	*G. ruber* (pink)	Marshall et al. [9]	Trap samples	+^b^	6		+	70		− with phosphate^b^
		Weinkauf et al. [20]	Fossil sediment				+*	NA	0	
+	*G. elongatus* (d’Orbigny, 1826)	This study	Trap samples	+	7	−	0*	7	+	
+	*N. dutertrei* (d’Orbigny, 1839)	Broecker and Clark [5]	Surface sediment				+	60		
+	*O. universa* d’Orbigny, 1839	Spero et al. [79]	Culture experiments				+	max. 700		
		Bijma et al. [10]	Culture experiments				+	800		
		Lombard et al. [11]	Culture experiments				+	270		
		Weinkauf et al. [20]	Fossil sediment				+*	NA	0	
+	*T. sacculifer* (Brady, 1877)	Broecker and Clark [5]	Surface sediment				+	60		
		Bijma et al. [25]	Culture experiments				+	500		
		Lombard et al. [11]	Culture experiments				+	495		+ with light irradience
		Naik et al. [80]	Fossil sediment	−^b^	NA		+*	45		
		Marshall et al. [9]	Trap samples	+^b^	5		+	90		
−	*G. bulloides* d’Orbigny, 1826	Barker and Elderfield [6]	Surface and fossil sediment	0^a^	16		+*	70		
		de Villiers [16]	Surface sediment						+^a^	
		Gonzalez-Mora et al. [76]	Fossil sediment	+^a^	8		+*^a^	NA	0^a^	
		Moy et al. [8]	Trap samples and surface sediment				+*	NA		
		Beer et al. [12]	Plankton net samples				+	120	0	
		Naik et al. [77]	Fossil sediment	−^b^	4		+*	NA	+	
		Aldridge et al. [15]	Plankton net samples	+	3	+	+	33	−	+ with growth potential, − with phosphate and nitrate
		Davis et al. [14]	Fossil sediment	+^a^	9					
		Mohan et al. [21]	Plankton net samples and surface sediment	0^a^	NA					
		This study	Trap samples	0	7	0	0*	7	−	
−	*G. inflata* (d’Orbigny, 1839)	Barker and Elderfield [6]	Surface and fossil sediment	0^a^	16		+*^a^	70		
		Weinkauf et al. [20]	Fossil sediment				+*	NA	0	
−	*G. puncticulata* (Deshayes, 1832)	Davis et al. [14]	Fossil sediment	+^a^	9					
−	*G. scitula* (Brady, 1882)	Weinkauf et al. [20]	Fossil sediment				+*	NA	0	
−	*G. truncatulinoides* (d’Orbigny, 1839)	Barker and Elderfield [6]	Surface and fossil sediment	0^a^	12		+*^a^	60		
		de Villiers [16]	Surface sediment						+^a^	
−	*N. incompta* (Cifelli, 1961)	Barker and Elderfield [6]	Surface and fossil sediment	0^a^	16		+*^a^	70		
		de Villiers [16]	Surface sediment						+^a^	
		Gonzalez-Mora et al. [76]	Fossil sediment	0^a^	6		0*^a^	NA	+^a^	
−	*N. pachyderma* (Ehrenberg, 1861)	Manno et al. [13]	Culture experiments	0	5		+	65		temperature moderates influence of carbonate ion
		Mohan et al. [21]	Plankton net samples and surface sediment	+^a^	NA					
−	*P. obliquiloculata* (Parker and Jones, 1862)	Broecker and Clark [5]	Surface sediment				+	60		

+ = positive correlation, − = negative correlation, 0 = no influence detected.

*Carbonate ion concentration indirectly inferred;

^a^Not explicitly tested, only implied;

^b^Attributed to collinearity;

^c^Unclear whether samples also included *G. elongatus* specimens;

^d^Unclear whether samples included only *G. ruber* (white) or *G. ruber* (pink) or both

The meta-analysis presented in Table 7 reveals that higher temperature and higher carbonate saturation, either determined directly or inferred from indirect proxies, seems to generally favor calcification. The effect of temperature is less frequently observed and confounded by collinearity [9] and potentially even interaction with carbonate saturation [13]. On the other hand, the effect of productivity and optimal growth conditions is ambiguous. Interestingly, these results seem to apply to symbiont-bearing and asymbiotic species alike, which would indicate that the presence of symbionts may affect the absolute values of calcification intensity and size–weight slope, but it does not modify the sign of the response of calcification intensity to the main candidate environmental parameters.

**Fig 13:**
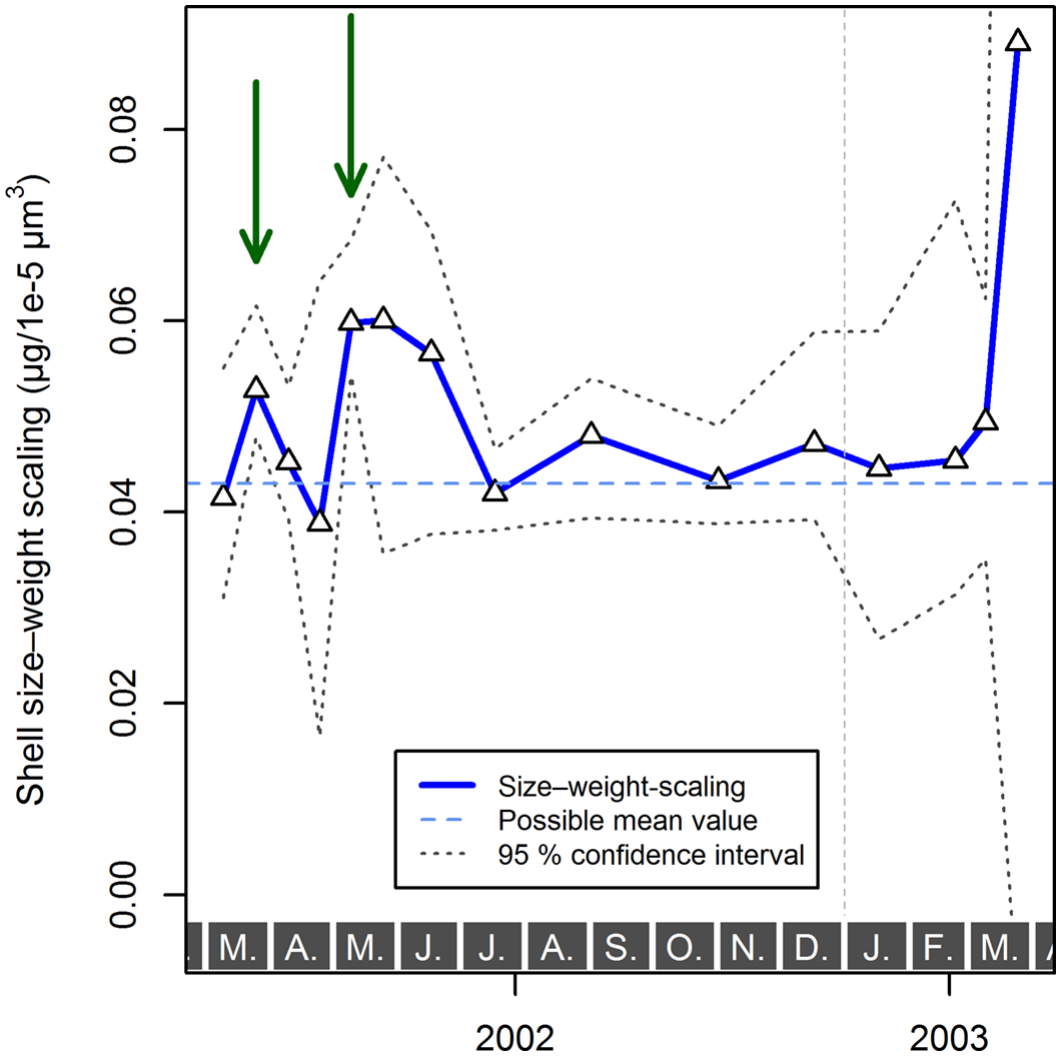
Estimated mean size–weight scaling and its 95% confidence interval of *Globigerinoides ruber* (white) and *Globigerinoides elongatus* sampled from March 2002 until April 2003 with sediment trap L1/K276 pooled together. Some samples either contained no or too few specimens to estimate the size–weight scaling or its confidence interval, or were not significant in the Kendall–Theil robust line fitting, and were thus not included in the analysis. A constant mean calcification in the synthetic taxon can only be assumed when disregarding two samples marked with the green arrows. The values generally appear too high from late winter to early summer. Gray boxes with letters at the bottom indicate months, the vertical, dashed, gray line marks the end of 2002.

The existence of species-specific response types and offsets in calcification intensity and size–weight scaling implies a potentially high sensitivity of the observed response type and strength to the accuracy of species identification. Our data provide first evidence for distinct patterns recorded by *G. ruber* (white) and *G. elongatus*. Traditionally, these species were often deliberately or unintentionally pooled for various purposes, assuming that *G. ruber* in its broad definition introduced by Parker [81] represents one biological species. In reality, as shown already by isotopic and trace-element analyses on *G. ruber* morphotypes [44], the two forms lumped within the broad concept of *G. ruber* (white) represent genetically distinct lineages with different ecological preferences [43]. Using the data in our study, we can simulate the effect of pooling *G. ruber* (white) and *G. elongatus* on calcification patterns in such a synthetic taxon. We observe that in the pooled dataset, already the first assumption needed to meaningfully interpret calcification intensity as approximated by AD is violated. In the pooled dataset, the size–weight scaling is not consistent across samples, giving the impression of the existence of steeper scaling slopes in winter (Fig 13). In reality, this simply reflects the times when *G. ruber* (white) was more abundant than *G. elongatus* (Fig 4). While the difference in size–weight scaling between both species is not significant when considering its entire variation, the combination of certain seasonal variability in their abundance and size–weight scaling can result in significant temporary outliers. These results imply that combining *G. ruber* (white) and *G. elongatus* into a single morphospecies for analyses of calcification intensities introduces an error that can neither be eliminated nor objectively quantified. Several of the investigations summarized in Table 7 may indeed be affected by this issue, explaining the lack of sensitivity of pooled *G. ruber* calcification data to temperature and carbonate ion as observed by Naik et al. [17] as well as the results by Beer et al. [12], which otherwise remains the only case documenting a negative correlation between calcification intensity and carbonate saturation in planktonic Foraminifera.

### Optimum Growth Conditions Reflected by Shell Size Distribution

The concept of growth under optimum conditions in planktonic Foraminifera has been originally devised to explain patterns of shell size distribution in surface sediments. This concept [18, 19, 82] is based on the observations that largest mean shell size occurs in samples where the analyzed species meet their environmental optimum. Since shell size data have been collected for the three species analyzed in this study, it is possible to test independently the assumption of using species flux in the sediment trap series as approximation of optimum growth conditions. If the concept by Hecht [18] holds, then shell size in *G. bulloides* should be largest during the late winter and spring when its abundance is highest and temperatures are lowest, closer to the optimum of the species, whose highest abundance in the sediment is to the north of the sediment trap region (Fig 2B, S1 File). For *G. ruber* (white) and *G. elongatus* the test will be more difficult because the preferences of the two species are not known from sediment distribution, because these two taxa have not been separated (Fig 2C). For both species combined, sediment distribution data indicate a warmer optimum habitat than the average conditions at the sediment trap site, but peak abundance in the studied series occurs in summer and early spring, indicating that the abundance of those species may have reacted to factors other than temperature (S1 File).

To test for correlation between environmental parameters and shell sizes in the three species analyzed, we use the same GLM models as applied to the calcification data. In *G. bulloides* we find Models (1) and (2) to be indistinguishable (ΔAIC_*c*_ = 1.1). Both models indicate that shell sizes in this species are correlated with water temperature and productivity (Table 6). As expected from the optimum growth model, shell size of *G. bulloides* is negatively correlated with SST (*ρ* = −0.142) and positively correlated with productivity (*ρ* = 0.306), reflecting generally cooler and more productive optimum conditions of the species (Fig 12B). A link of shell size with flux could not be observed however. This seems to reflect the asymmetry of flux between spring and autumn in 2002 and underlines the observation of a large interannual variability at the studied site [39]. It seems that in such situations the pattern of flux, rather than the absolute values, would be a better estimate of optimum growth conditions. For an analysis of the two species of *Globigerinoides* we find Model (4) to be the most informative, with the other models being clearly inferior (Model (3): ΔAIC_*c*_ = 11.7, Model (2): ΔAIC_*c*_ = 160.1, Model (1): ΔAIC_*c*_ = 164.2). However, this model implies that none of the environmental parameters nor flux has significantly affected the size distribution (Table 8), confirming the observation that shell sizes of both species remained rather similar throughout the studied period (Fig 5).

**Table 8:** Results of the most informative generalized linear Model (4) for the shell size of *Globigerinoides* species from trap L1/K276 in the North Atlantic.

	Standard error	*t*-value	*p*-value
Intercept	54.6034	2.618	.009
SST	2.257	1.230	.219
Chl. *a*	79.604	1.220	.223
Biospecies	88.803	1.724	.085
Shell flux	0.800	1.202	.230
SST × biospecies	3.740	−1.607	.109
Chl. *a* × biospecies	126.063	0.414	.679
Shell flux × biospecies	1.294	0.961	.337

The model implies that shell size is not influenced by any factor monitored.

The distribution of shell sizes in the three investigated species thus does not lend strong support to the hypothesis that optimum growth conditions result in largest shell sizes. Alternatively, it may be that absolute abundance as used in this study is not the best descriptor of optimum growth conditions and relative abundance in relationship to all planktonic Foraminifera in the analyzed samples, as used by Hecht [18], is more appropriate. To this end, we carried out the same tests but used relative abundance data from Storz et al. [39]. Since these were not available for *G. ruber* (white) and *G. elongatus* separately, we only carried out an analysis according to Model (2) for both *Globigerinoides* species. The results are virtually similar to those when using absolute abundance: no significant relationship between relative abundance and size could be found. The same applies even when the analyses of calcification intensity are repeated with relative abundance instead of flux (S1 File). Thus, we conclude that across the range of environmental conditions represented in our study, shell size does not seem to reflect optimum growth conditions, implying that perhaps even the interpretation of the observed relationships between optimum growth conditions and calcification intensity have to be interpreted with caution. The possibility that calcification is not related to optimum growth conditions or optimum growth conditions are difficult to approximate by abundance could explain the inconsistent reaction of calcification to this parameter observed by our and earlier studies (Table 7). We must note, however, that the range of environmental change observed during the year-long study presented here is much smaller than the range of environmental change in the studies which established the relationship between shell size and optimum growth conditions in planktonic Foraminifera [18, 19, 82]. It is thus possible that the effect size in our study was too small to detect any significant correlation, and that over larger environmental gradients correlations between calcification intensity and optimum environmental conditions do exist, which could also explain the regarding inconsistency in Table 7.

## Supporting Information

S1 Table Carbonate equilibrium system of the sea water. Long-term data for the catchment area of sediment trap L1/K276 and data for site ESTOC during the sampling period.
(XLSX)

S1 File Results from additional analyses. Microbalance accuracy. Correlation between shell flux and environment. Size–weight scaling. Correlation between size–weight scaling and calcification intensity. Correlation between shell flux and shell calcification intensity. Results of tests for normality and unimodality of shell size and shell calcification intensity. Assessment of the suitability of linear vs. exponential models for the size–weight scaling of planktonic Foraminifera shells. Comparison between the AD and MAD approach to quantify shell calcification intensity. Results of generalized linear models for shell size and shell calcification intensity using relative abundance instead of shell flux.
(PDF)
