# Supplementary material for: Seasonal Variation in Shell Calcification of Planktonic Foraminifera in the NE Atlantic Reveals Species-Specific Response to Temperature, Productivity, and Optimum Growth Conditions
Source: PLoS One. 2016 Feb 9;11(2):e0148363. doi: 10.1371/journal.pone.0148363 (PMC4747607; doi:10.1371/journal.pone.0148363)
Supplement: S1 File — Microbalance accuracy. Correlation between shell flux and environment. Size–weight scaling. Correlation between size–weight scaling and calcification intensity. Correlation between shell flux and shell calcification intensity. Results of tests for normality and unimodality of shell size and shell calcification intensity. Assessment of the suitability of linear vs. exponential models for the size–weight scaling of planktonic Foraminifera shells. Comparison between the AD and MAD approach to quantify shell calcification intensity. Results of generalized linear models for shell size and shell calcification intensity using relative abundance instead of shell flux. (PDF) [file pone.0148363.s002.pdf]

Electronic supplements to “Seasonal Variation in Shell Calcification of Planktonic Foraminifera in the NE Atlantic Reveals Species-Specific Response to Temperature, Productivity, and Optimum Growth Conditions”
PLOS ONE

Manuel F. G. Weinkauf[1,2,#a,*], José G. Kunze[1], Joanna J. Waniek[3], Michal Kučera[2]

1 Department of Geosciences, Eberhard–Karls University Tübingen, Tübingen, Germany, **2** MARUM, University Bremen, Bremen, Germany, **3** Marine Chemistry Section, Leibniz Institute for Baltic Sea Research Warnemünde, Rostock, Germany

#a Current Address: Department of Earth Sciences, University of Geneva, Geneva, Switzerland

* Manuel.Weinkauf@unige.ch

1 Microbalance accuracy

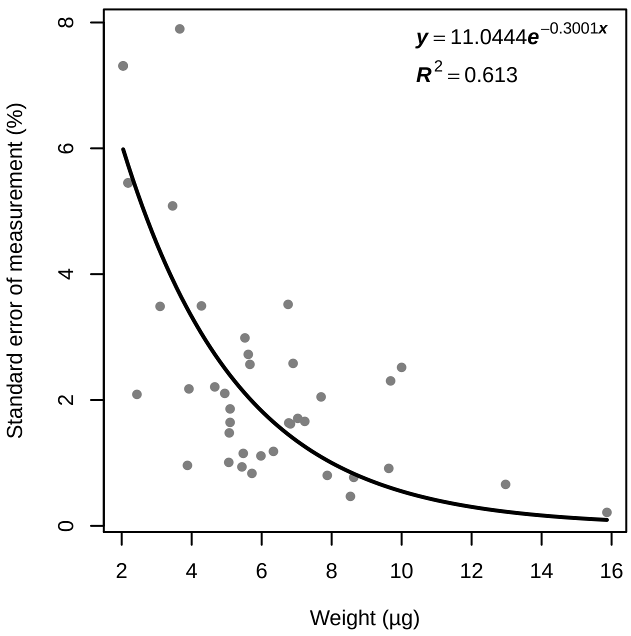

Figure S1. Relative standard error of weight measurements as function of mean weight of the specimen, using a Mettler Toledo UMX 2 microbalance. The measurement error can reach relatively high values for weights below 4 µg, and exponentially decreases with increasing weight.

2 Foraminiferal shell abundance

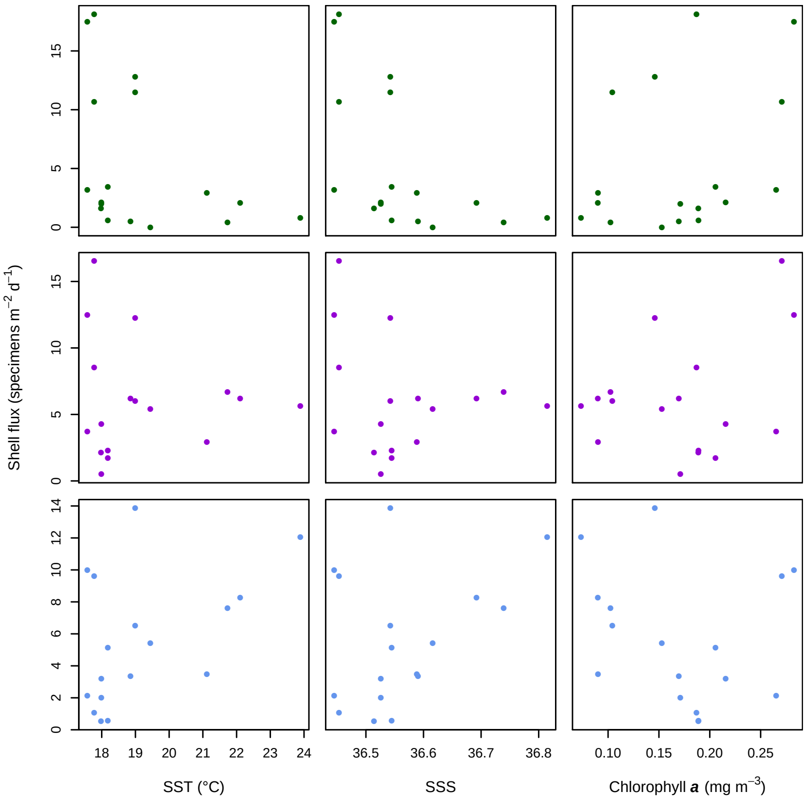

Figure S2. Relationship between shell flux and environmental conditions in *Globigerina bulloides* (top row), *Globigerinoides ruber* (white) (middle row), and *Globigerinoides elongatus* (bottom row), sampled from March 2002 until April 2003 with sediment trap L1/K276. Environmental data include sea surface temperature (SST; NOAA/OAR/ESRL PSD, `http://www.esrl.noaa.gov/psd/`), sea surface salinity (SSS; [1]), and surface chlorophyll *a* [2].

3 Foraminiferal shell size and calcification

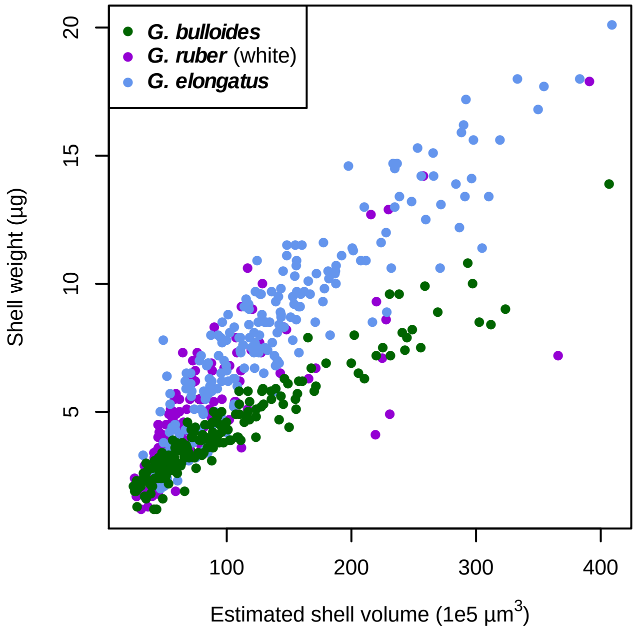

Figure S3. Relationship between shell size (approximated as volume) and weight in *Globigerina bulloides*, *Globigerinoides ruber* (white), and *Globigerinoides elongatus* sampled from March 2002 until April 2003 with sediment trap L1/K276. No strong effect of gametogenetic calcification with size is observable, although shells below 5 000 000 µm^3 seem to show a slightly less intense calcification (especially in *G. elongatus*). The residuals of a robust linear regression in no species showed a significant bimodality ($p_{bulloides} = .694$, $p_{ruber} = .897$, $p_{elongatus} = .963$, [3]), as would be expected when part of the population would be influenced by intense gametogenetic calcification. *Globigerina bulloides* shows a much smaller size–weight scaling slope than the two *Globigerinoides* species.

Table S1. Tests for normality and unimodality of size and calcification intensity (expressed as area density) data of *Globigerina bulloides*, *Globigerinoides ruber* (white), and *Globigerinoides elongatus* sampled from March 2002 until April 2003 with sediment trap L1/K276. Size data (Feret diameter) had been log-transformed before the test for normality of distribution. Provided are the p-values of a Shapiro–Wilk test for normality of distribution [4] and of Hartigan's dip test for unimodality [3]. In nearly all cases, both shell size and calcification intensity are unimodally normally distributed, indicating the existence of only one population per sample.

	G. bulloides		*G. ruber* (white)		*G. elongatus*	
Sample	Normality	Unimodality	Normality	Unimodality	Normality	Unimodality
**Shell size**						
2	0.504	0.474	0.360	0.342	0.441	1.000
3	0.152	0.136	0.034	0.027	0.403	0.025
4	0.198	0.571	0.063	0.013	0.352	0.817
5	0.066	0.951	0.078	0.021	NA	NA
6	0.978	0.941	0.117	0.030	0.019	0.991
7	0.505	0.910	0.861	0.326	0.185	0.581
8	0.657	0.698	0.220	0.355	0.748	0.607
9	0.851	0.483	0.066	0.443	0.625	0.985
10	0.030	0.001	NA	NA	0.602	0.335
11	0.209	1.000	0.301	0.224	0.153	0.998
12	NA	NA	0.201	0.356	0.244	0.888
13	NA	NA	0.883	1.000	0.972	0.630
14	NA	NA	0.024	0.000	NA	NA
15	0.034	0.005	0.891	1.000	0.157	0.028
16	0.991	1.000	0.847	1.000	0.614	0.731
17	0.349	0.176	NA	NA	0.085	0.028
18	0.058	1.000	0.001	1.000	NA	NA
**Calcification intensity**						
2	0.266	0.781	0.479	0.167	0.250	1.000
3	0.279	0.437	0.049	0.652	0.096	0.499
4	0.401	0.272	0.000	0.010	0.239	0.516
5	0.110	0.561	0.033	0.020	NA	NA
6	0.000	0.992	0.087	0.134	0.908	0.977
7	0.155	0.211	0.762	0.866	0.253	0.631
8	0.221	0.107	0.043	0.483	0.468	0.619
9	0.198	0.133	0.111	0.839	0.222	0.868
10	0.486	0.528	NA	NA	0.006	0.825
11	0.683	1.000	0.664	1.000	0.050	0.845
12	NA	NA	0.626	0.510	0.885	0.928
13	NA	NA	0.323	0.052	0.135	0.590
14	NA	NA	0.024	0.000	NA	NA
15	0.607	0.554	0.379	1.000	0.317	0.911
16	0.294	0.134	0.800	1.000	0.146	0.552
17	0.398	1.000	NA	NA	0.633	0.249
18	0.076	1.000	0.001	1.000	NA	NA

Table S2. Summary of a comparison of the coefficient of determination between a linear model and an exponential model to describe the size–weight relationship in *Globigerina bulloides*, *Globigerinoides ruber* (white), and *Globigerinoides elongatus* sampled from March 2002 until April 2003 with sediment trap L1/K276. Provided are N number of samples for which comparisons were made, in how many of those the R^2-value was significantly increased by the exponential function, and in how many cases the exponential model decreased the coefficient of determination. While the exponential function described the data better in approximately 14 % of all cases, it decreased the fit in nearly 35 % of cases. We thus conclude that the approximated transformation of shell cross-sectional area into shell volume was sufficient to linearize the relationship.

	N	Sig. increase	Decrease
G. bulloides	16	1	6
G. ruber (white)	17	3	7
G. elongatus	17	3	4
Total	50	7	17

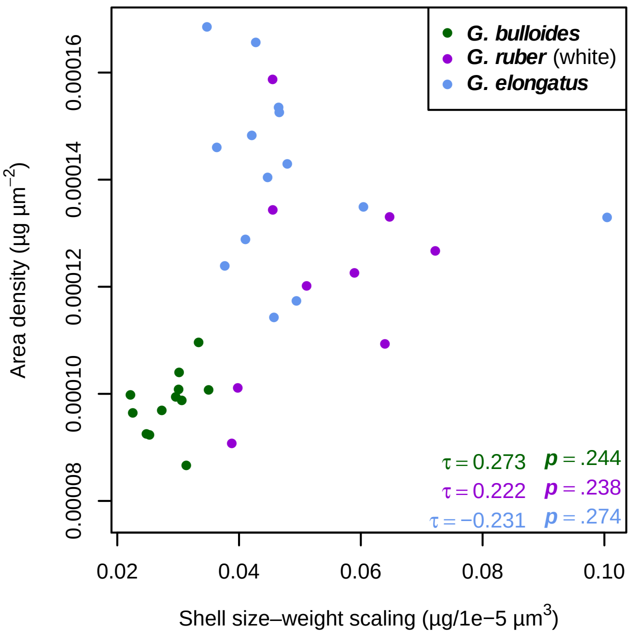

Figure S4. Calcification intensity (expressed as area density) as function of the size–weight scaling slope in *Globigerina bulloides*, *Globigerinoides ruber* (white), and *Globigerinoides elongatus* sampled from March 2002 until April 2003 with sediment trap L1/K276. The correlation coefficient τ and the p-value for the significance of the correlation are provided. No significant relationship between the two parameters could be detected in any species.

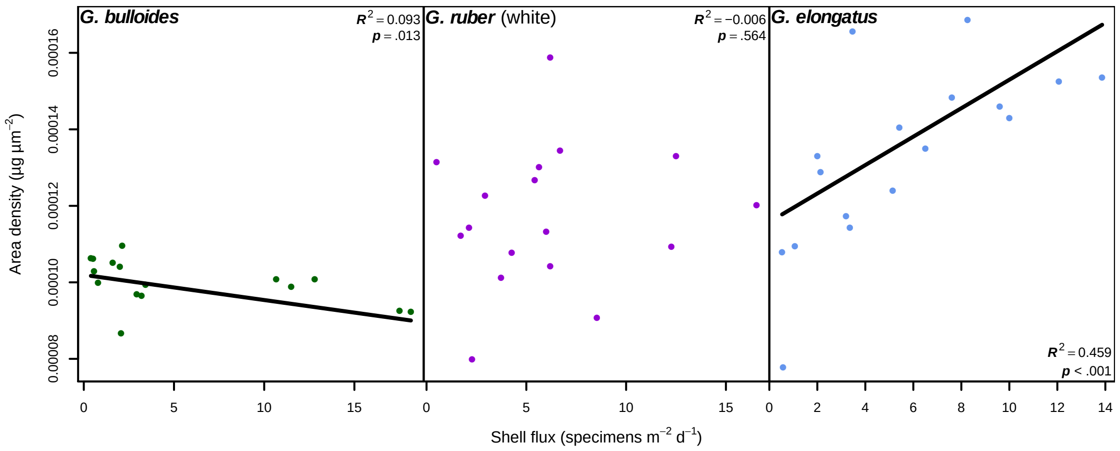

Figure S5. Relationship between shell flux and average area density of *Globigerina bulloides*, *Globigerinoides ruber* (white), and *Globigerinoides elongatus*, sampled from March 2002 until April 2003 with sediment trap L1/K276. Raw data points and the regression line of a Kendall–Theil robust line fitting are shown. While there is a clear correlation between abundance and shell calcification in *G. elongatus*, it is questionable in the two other species. In *G. bulloides* such a relationship is still significant, but the R^2-value is very low, indicating that the majority of the observed variance can be attributed to other sources. Furthermore, the observed relationship is negative, indicating reduced calcification in more suitable environments. In *G. ruber* (white) no such relationship can be observed at all.

4 Comparison between area density and mean area density

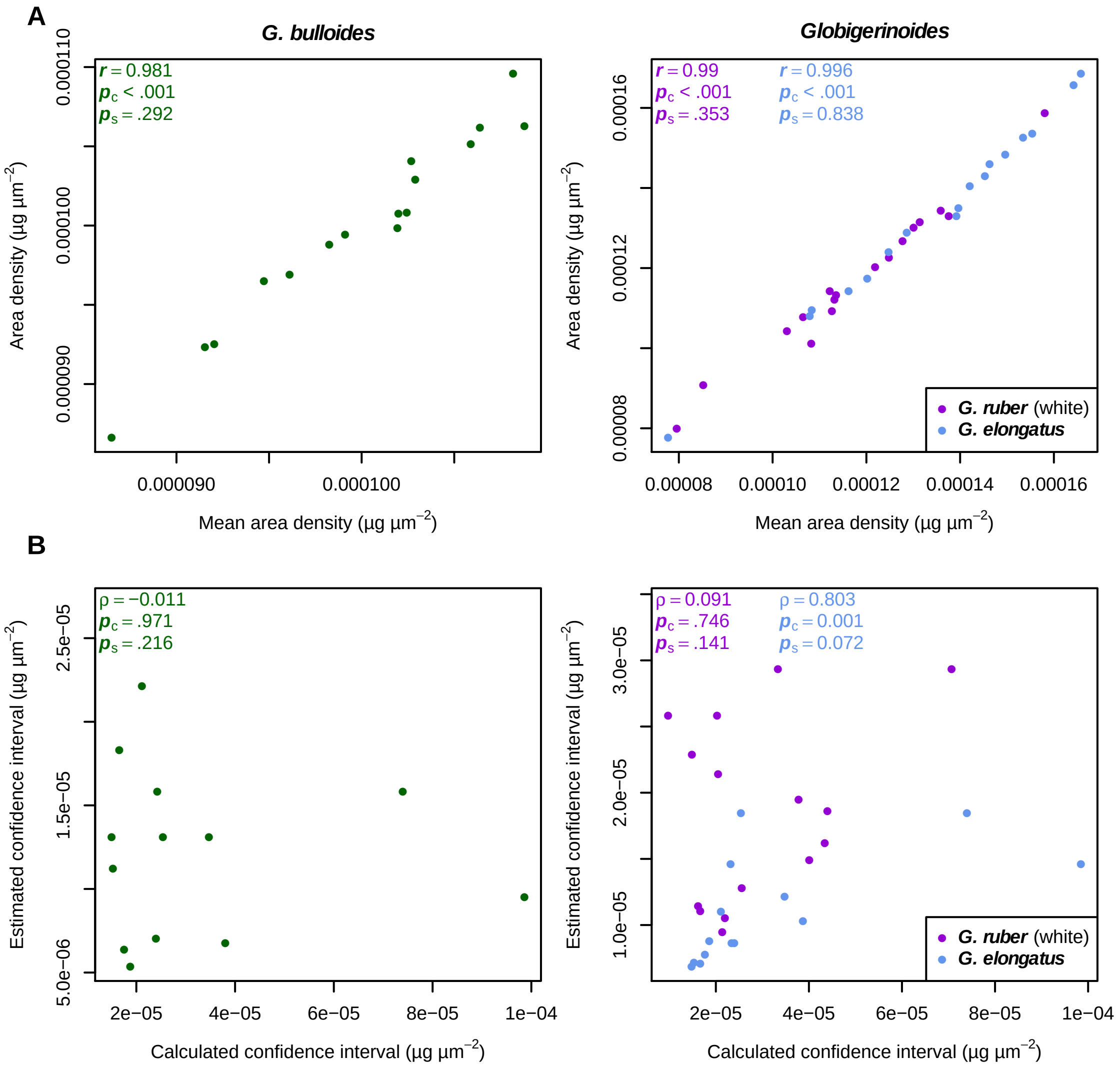

Figure S6. Comparison between mean area density (MAD, [5]) and mean of individual area density values (AD) per sample of *Globigerina bulloides*, *Globigerinoides ruber* (white), and *Globigerinoides elongatus*, sampled from March 2002 until April 2003 with sediment trap L1/K276. The correlation coefficients r of a Pearson product-moment correlation and ρ of a Spearman rank-order correlation (including the corresponding p_c-value) and the p_s-value for the slope of the correlation being different from one (calculated with the R-package “smatr” v. 3.4-3, [6]) is also provided. (A) In all species the correlation between MAD and AD is significant with the slope never being significantly different from one, indicating a very high agreement in calculated calcification intensities between both methods. (B) The estimated confidence intervals for the MAD approach are in most cases not significantly correlated with the bootstrapped confidence intervals per sample. The estimation approach systematically tends to underestimate the true confidence intervals.

5 Correlation of shell size and calcification with relative abundances

Table S3. Results of a generalized linear model for the shell calcification intensity of *Globigerinoides* species from trap L1/K276 in the North Atlantic. The model implies that shell calcification in that taxon is influenced by temperature and productivity, but not correlated to relative abundance. Relative abundances were derived from Storz et al. [7].

	Standard error	t-value	p-value
Intercept	4.304×10^{-5}	−3.635	<0.001
SST	2.111×10^{-6}	6.540	<0.001
Chl. *a*	3.789×10^{-5}	4.007	<0.001
Relative abundance	2.094×10^{-7}	−1.434	0.152

Table S4. Results of a generalized linear model for the shell size of *Globigerinoides* species from trap L1/K276 in the North Atlantic. The model implies that shell size in that taxon is influenced by productivity, but not correlated to relative abundance. Relative abundances were derived from Storz et al. [7].

	Standard error	t-value	p-value
Intercept	52.253	2.722	0.007
SST	2.398	1.706	0.088
Chl. *a*	63.118	2.540	0.011
Relative abundance	0.257	−0.627	0.531

Table S5. Results of a generalized linear model for the shell calcification intensity of *Globigerina bulloides* from trap L1/K276 in the North Atlantic. The model implies that shell calcification in that taxon is not influenced by any parameter. Relative abundances were derived from Storz et al. [7].

	Standard error	t-value	p-value
Intercept	3.624×10^{-5}	4.384	<0.001
SST	1.496×10^{-6}	−1.556	0.121
Chl. *a*	3.329×10^{-5}	−1.869	0.063
Relative abundance	3.164×10^{-7}	−1.900	0.059

Table S6. Results of a generalized linear model for the shell size of *Globigerina bulloides* from trap L1/K276 in the North Atlantic. The model implies that shell size in that species is influenced by productivity, but not correlated to relative abundance. Relative abundances were derived from Storz et al. [7].

	Standard error	*t*-value	*p*-value
Intercept	123.579	0.351	0.726
SST	5.109	1.520	0.130
Chl. *a*	115.525	3.205	0.002
Relative abundance	1.077	−0.047	0.962

References

1. Good SA, Martin MJ, Rayner NA. EN4: Quality controlled ocean temperature and salinity profiles and monthly objective analyses with uncertainty estimates. J Geophys Res, Oceans. 2013; 118(12): 6704–6716. doi: 10.1002/2013JC009067

2. Yoder J, Kennelly M. Live access to US JGOFS SMP data: Global SeaWiFS chlorophyll. U.S. JGOFS; 2005. Available from: `http://usjgofs.whoi.edu/las?dset=Ocean+Color/Global+SeaWiFS+chlorophyll+1997-2004`.

3. Hartigan JA, Hartigan PM. The dip test of unimodality. Ann Stat. 1985; 13: 70–84. doi:10.1214/aos/1176346577

4. Shapiro SS, Wilk MB. An analysis of variance test for normality (complete samples). Biometrika. 1965; 52: 591–611. doi: 10.1093/biomet/52.3-4.591

5. Weinkauf MFG, Moller T, Koch MC, Kučera M. Calcification intensity in planktonic Foraminifera reflects ambient conditions irrespective of environmental stress. Biogeosciences. 2013; 10: 6639–6655. doi: 10.5194/bg-10-6639-2013

6. Warton DI, Duursma RA, Falster DS, Taskinen S. smatr 3 – an R package for estimation and inference about allometric lines. Methods Ecol Evol. 2012; 3: 257–259. doi: 10.1111/j.2041-210X.2011.00153.x

7. Storz D, Schulz H, Waniek JJ, Schulz-Bull DE, Kučera M. Seasonal and interannual variability of the planktic foraminiferal flux in the vicinity of the Azores Current. Deep-Sea Res, Part I. 2009; 56: 107–124. doi: 10.1016/j.dsr.2008.08.009
